# Synthesis and crystal structures of tetra­meric [2-(4,4-dimethyl-2-oxazolin-2-yl)anilido]sodium and tris­[2-(4,4-dimethyl-2-oxazolin-2-yl)anilido]ytterbium(III)

**DOI:** 10.1107/S2056989020005034

**Published:** 2020-04-21

**Authors:** Leah Gajecki, Brendan Twamley, David J. Berg

**Affiliations:** a Department of Chemistry, University of Victoria, PO Box 1700 Stn CSC, Victoria, BC V8W 2Y2, Canada; b School of Chemistry, Trinity College Dublin, University of Dublin, Dublin 2, Ireland

**Keywords:** crystal structure, synthesis, ytterbium, oxazoline, amide

## Abstract

Reaction of 2-(4,4-dimethyl-2-oxazolin-2-yl)aniline (H_2_-**L1**) with one equivalent of Na[N(SiMe_3_)_2_] in toluene afforded pale-yellow crystals of tetra­meric poly[bis­[μ_3_-2-(4,4-dimethyl-2-oxazolin-2-yl)anilinido][μ_2_-2-(4,4-dimethyl-2-oxa­zolin-2-yl)aniline]tetra­sodium(I)], [Na_4_(C_11_H_13_N_2_O)_4_]_*n*_ or [Na_4_(H-**L1**)_4_]_*n*_ (**2**), in excellent yield. Subsequent reaction of [Na_4_(H-**L1**)_4_]_*n*_ (**2**) with 1.33 equivalents of anhydrous YbCl_3_ in a 50:50 mixture of toluene–THF afforded yellow crystals of tris­[2-(4,4-dimethyl-2-oxazolin-2-yl)anilinido]ytterbium(III), [Yb(C_11_H_13_N_2_O)_3_] or Yb(H-**L1**)_3_ (**3**) in moderate yield. Direct reaction of three equivalents of 2-(4′,4′-dimethyl-2′-oxazolin­yl)aniline (H_2_-**L1**) with Yb[N(SiMe_3_)_2_]_3_ in toluene resulted in elimination of hexa­methyl­disilazane, HN(SiMe_3_)_2_, and produced Yb(H-**L1**)_3_ (**3**) in excellent yield. The structure of **2** consists of tetra­meric Na_4_(H-**L1**)_4_ subunits in which each Na^+^ cation is bound to two H-**L1** bridging bidentate ligands and these subunits are connected into a polymeric chain by two of the four oxazoline O atoms bridging to Na^+^ cations in the adjacent tetra­mer. This results in two 4-coordinate and two 5-coordinate Na^+^ cations within each tetra­meric unit. The structure of **3** consists of a distorted octa­hedron where the bite angle of ligand **L1** ranges between 74.72 (11) and 77.79 (11) degrees. The oxazoline (and anilide) N atoms occupy meridional sites such that for one ligand an anilide nitro­gen is *trans* to an oxazoline nitro­gen while for the other two oxazoline N atoms are *trans* to each other. This results in a significantly longer Yb—N(oxazoline) distance [2.468 (3) Å] for the bond *trans* to the anilide compared to those for the oxazoline N atoms *trans* to one another [2.376 (3), 2.390 (3) Å].

## Abstract 2

The reaction of 2-(4′,4′-dimethyl-2′-oxazolin­yl)aniline (H_2_-**L1**) with Na[N(SiMe_3_)_2_] afforded colourless crystals of tetra­meric Na_4_(H-**L1**)_4_ (**2**). Reaction of either Na_4_(H-**L1**)_4_ (**2**) with YbCl_3_ or reaction of H_2_-**L1** with Yb[N(SiMe_3_)_2_]_3_ afforded yellow crystals of the highly distorted octa­hedral complex Yb(H-**L1**)_3_ (**3**).

## Chemical context   

**Figure 1:**
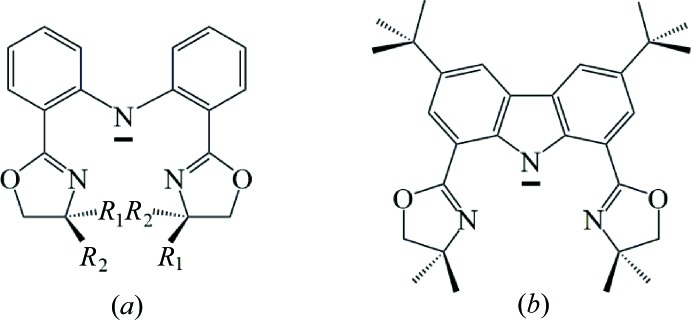
Related ligand types: (*a*) oxazoline-di­phenyl­amides and (*b*) carbazolide-bis­(oxazolines).

**Figure 2:**
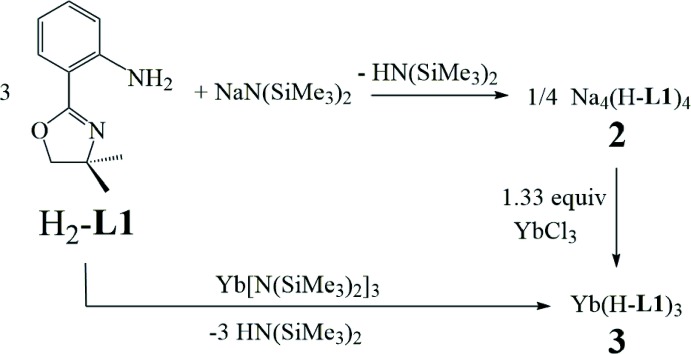
Synthetic routes to [Na_4_(H-**L1**)_4_]_*n*_ (**2**) and Yb(H-**L1**)_3_ (**3**) used in this work.

The parent ligand 2-(4′,4′-dimethyl-2′-oxazolin­yl)aniline (H_2_-**L1**), easily prepared in high yield using established procedures (Gossage, 2009 ▸), has been used as a precursor to biologically active quinilones [see, for example, Hong *et al.* (2018 ▸)] and to make catalytically active transition-metal complexes (Saiyed *et al.*, 2011 ▸; Resanović *et al.*, 2011 ▸; Decken *et al.*, 2005 ▸). There are many examples of transition metals containing N-substituted variants of **L1**, either as neutral ligands (H*R*-**L1**) or as deprotonated anilido anions (*R*-**L1** ^−^). However, the only example of a transition-metal structure containing the deprotonated and unsubstituted anilido parent ligand (H-**L1** ^−^) is an Ru carbonyl hydride dimer (Cabeza *et al.*, 2006 ▸). No lanthanide complexes of this ligand have been reported, although there are several related lanthanide and yttrium complexes bearing oxazoline groups *ortho* to an anilido-like anionic centre. These complexes fall into two main ligand frameworks: di­phenyl­amido ligands bearing *ortho*-oxazoline functionality (Fig. 1 ▸ *a*: Bennett *et al.*, 2013 ▸, 2014 ▸; Liu *et al.*, 2013 ▸) or carbazolide-bis­(oxazolines) (Fig. 1 ▸ *b*: Zou *et al.*, 2011 ▸, 2013 ▸). The crystal structure of the tetra­meric sodium salt of this ligand, [Na_4_(H-**L1**)_4_]_*n*_ (**2**), and its 6-coordinate, monomeric ytterbium complex, Yb(H-**L1**)_3_ (**3**) are reported in this communication. The ytterbium complex **3** can be prepared by either the salt metathesis reaction between **2** and YbCl_3_ or by the acid–base (protonolysis) reaction of Yb[N(SiMe_3_)_2_]_3_ with three equivalents of H_2_-**L1**. The yields and purity of **3** are better for the protonolysis reaction (Fig. 2 ▸).
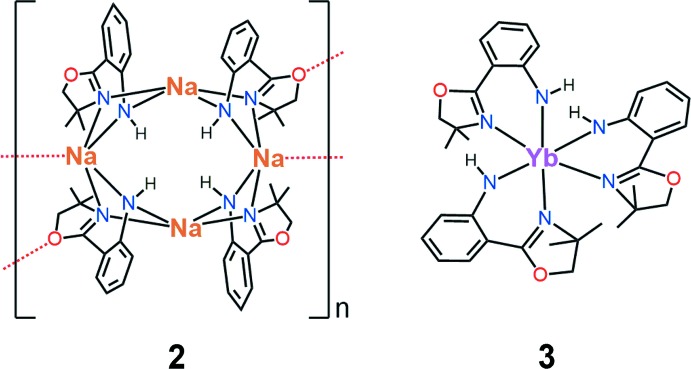


## Structural commentary   

**Figure 3:**
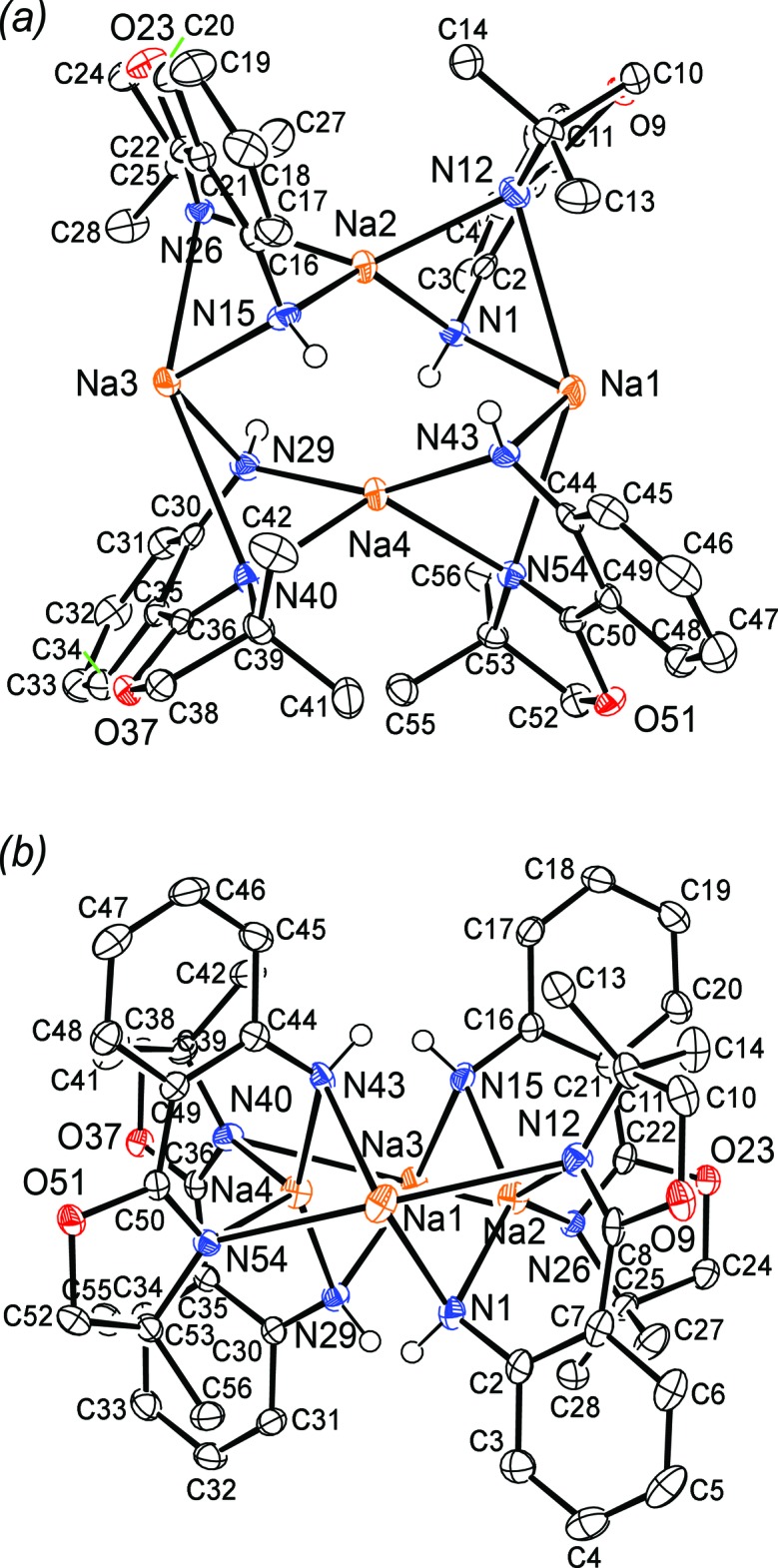
Mol­ecular structure of polymeric [Na_4_(H-**L1**)_4__*n*_ (**2**): (*a*) asymmetric unit, top view and (*b*) asymmetric unit, side view. Probability ellipsoids are at 50% and hydrogen atoms are omitted for clarity (except the aniline NH).

**Figure 4:**
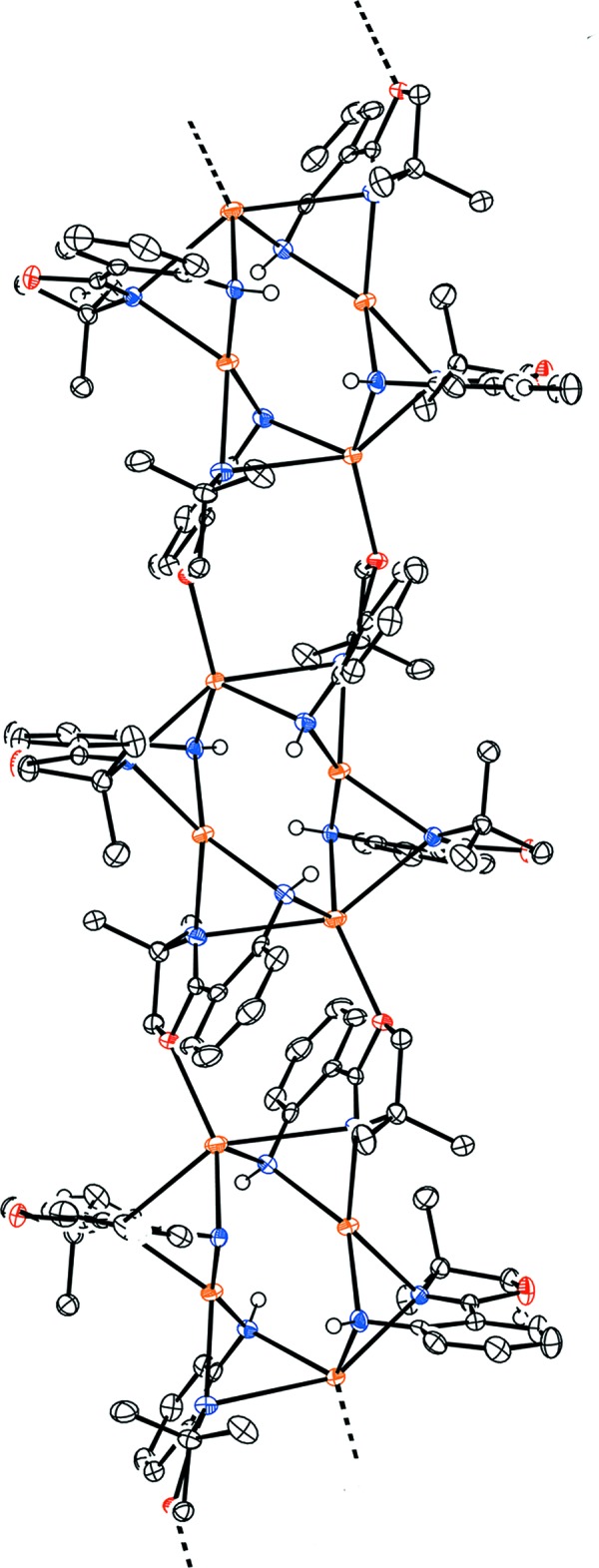
Mol­ecular structure of polymeric [Na_4_(H-**L1**)_4_]_*n*_ (**2**) showing the polymeric chain structure for three asymmetric units (top view). Probability ellipsoids are at 50% and hydrogen atoms are omitted for clarity (except the aniline NH).

The structure of **2** consists of tetra­meric Na_4_(H-**L1**)_4_ subunits in which each Na^+^ cation is bound to two H-**L1** bridging bidentate ligands (Fig. 3 ▸ *a* and 3*b*). The tetra­meric subunits are connected into polymeric chains by two of the four oxazoline oxygens (O9 and O37) bridging to Na^+^ cations (Na1 and Na3, respectively) in the adjacent tetra­mer (Fig. 4 ▸). This results in two 4-coordinate (Na2, Na4) and two 5-coordinate (Na1, Na3) Na^+^ cations within each tetra­meric unit. There are only four examples of an oxazoline ligand bonding through the *oxygen* atom and in all cases this involves an electropositive metal ion [Li: Pawilkowski *et al.* (2009 ▸) and Mukherjee *et al.* (2010 ▸); Na: Zou *et al.* (2013 ▸); Nd: Kanbur *et al.* (2018 ▸)]. Significant bond lengths and angles for **2** are collected in Table 1 ▸. The bridging Na—O(oxazoline) distances of 2.4003 (15) and 2.4099 (14) Å compare well the Na—O(oxazoline) distance of 2.432 (2) Å in NaCzx [Czx = 1,8-bis­(4′,4′-di­methyl­oxazolin-2′-yl)-3,6-di-*tert*-butyl­carbazole anion; Zou *et al.*, 2013 ▸]. The Na—N distances from the anilide N center to the 4- and 5-coordinate Na ions are essentially the same [2.3411 (18)–2.3701 (18) *versus* 2.3360 (18)–2.3611 (17) Å, respectively]. In sharp contrast, the distance between the oxazoline N and the Na ions is much shorter for the 4-coordinate Na centres [non-O-bridging oxazoline: 2.3626 (17), 2.3396 (17); O-bridging oxazoline: 2.4407 (17), 2.4035 (17) Å] than for the 5-coordinate Na [non-O-bridging oxazoline: 2.5515 (16), 2.7348 (17); O-bridging oxazoline: 2.9532 (18), 3.0327 (18) Å]. It is clear that the oxazoline nitro­gen is much more weakly coordinating to the 5-coordinate Na cation. In fact, for the O-bridging oxazoline, this distance is so long that it is debatable whether there is a significant bonding inter­action. However, this result is consistent with localization of electron density on the bridging oxygen at the expense of the nitro­gen atom in the same oxazoline ring.

**Figure 5:**
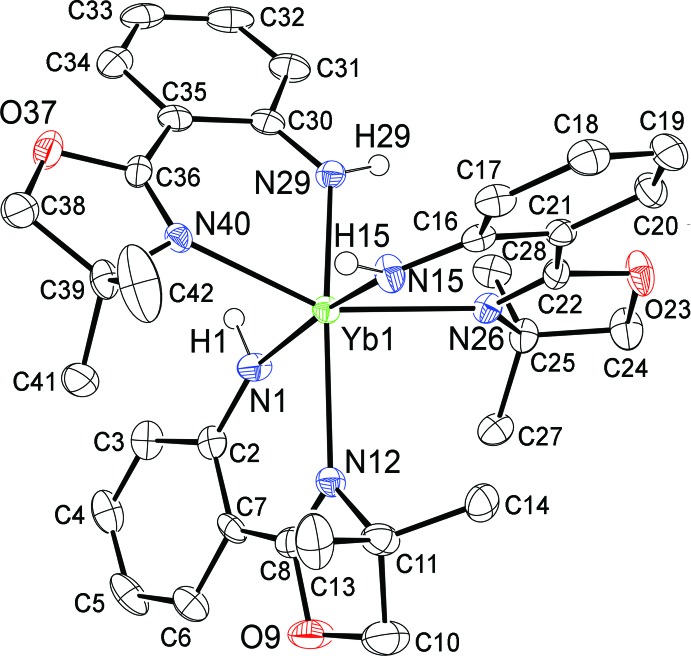
Mol­ecular structure of Yb(H-**L1**)_3_ (**3**). Probability ellipsoids are at 50% and hydrogen atoms are omitted for clarity (except the aniline NH).

The structure of Yb(H-**L1**)_3_ (**3**) is a distorted octa­hedron where all three H-**L1** ligands are distinct (Fig. 5 ▸). Significant geometric parameters for this compound are given in Table 2 ▸. The bite angles of the H-**L1** ^−^ ligand range from 74.72 (11)–77.79 (11)°, which sits between that of the di­phenyl­amido-oxazoline [see Fig. 1 ▸ *a*: range 69.97 (11)–80.1 (5)°, median 74.3°; Bennett *et al.* (2013 ▸, 2014 ▸); Liu *et al.* (2013 ▸)] and carbazolide-bis­(oxazoline) [see Fig. 1 ▸ *b*: range 77.99 (4)–81.18 (10)°, median 80.3°; Zou *et al.* (2011 ▸, 2013 ▸)] ligands. The oxazoline (and anilide) nitro­gens occupy meridional sites such that for one ligand an anilide nitro­gen is *trans* to an oxazoline nitro­gen while the other two oxazoline nitro­gens are *trans* to each other. This results in a significantly longer Yb—N(oxazoline) distance [2.468 (3) Å] for the bond *trans* to the anilide compared to those for the oxazolines *trans* to one another [2.376 (3), 2.390 (3) Å]. The Yb—N(anilide) distances [2.234 (3)–2.260 (3) Å] show less variation although the Yb—N(anilide) distance *trans* to the oxazoline N atom is slightly shorter than those *trans* to each other. Overall, this is consistent with a stronger *trans* influence for the anionic anilide nitro­gens as might be expected. The torsion angles representing twisting from coplanarity of the oxazoline and benzene units all fall between −9.3 (6) and +8.4 (6)° so the distortions from planarity of the H-**L1** ligands in **3** are relatively small.

## Supra­molecular features   

**Figure 6:**
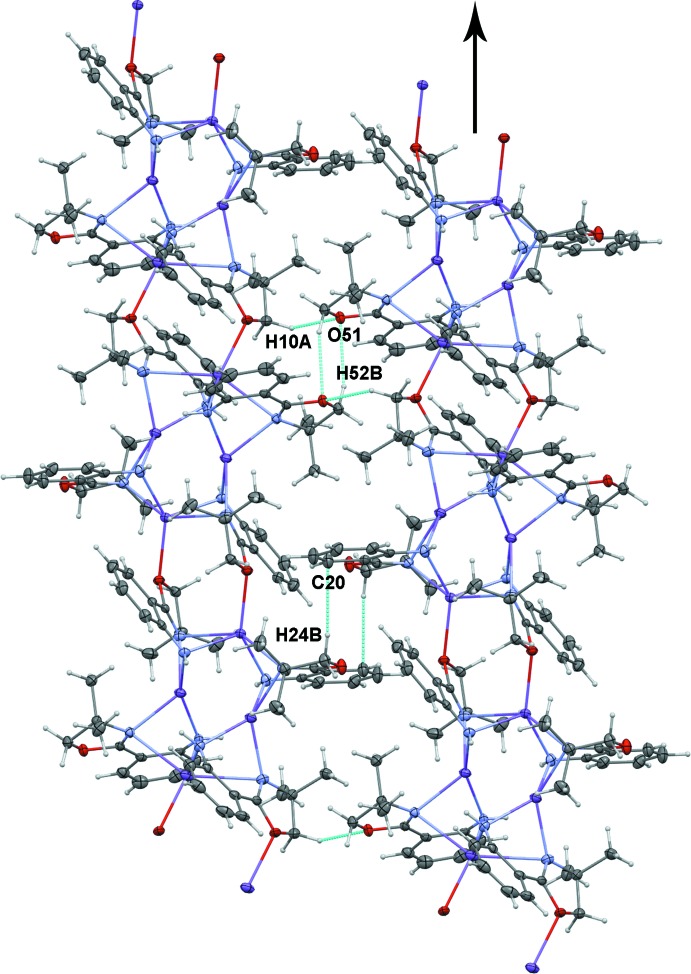
Close contacts between polymeric chains of Na_4_(H-**L1**)_4_ (**2**): inter­chain contacts consisting of C—H⋯O and C—H⋯π inter­actions are shown in teal; the chain direction is indicated by the arrow.

**Figure 7:**
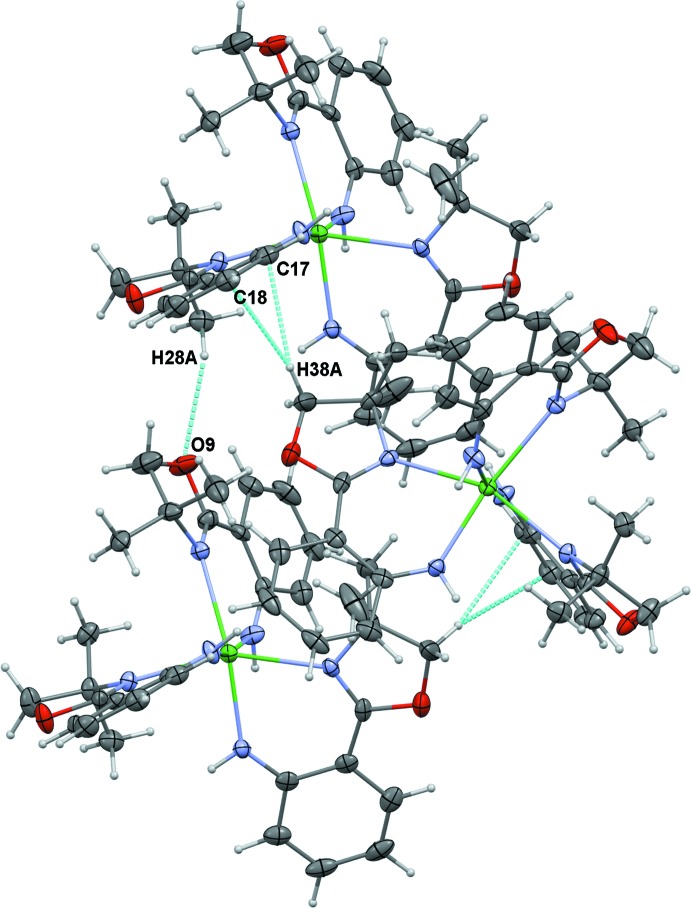
Close contacts between mol­ecules of Yb(H-**L1**)_3_ (**3**) in the solid state: C—H⋯O and C—H⋯π inter­actions between adjacent mol­ecules are shown in teal.

The structure of **2** consists of polymeric chains of Na_4_(H-**L1**)_4_ subunits connected through bridging oxazoline oxygen atoms (Fig. 4 ▸). There are two different types of close contacts between adjacent polymer chains through the non-O-bridging oxazoline rings (Fig. 6 ▸, Table 3 ▸). One type involves the close approach of one H atom of two different oxazoline CH_2_ groups to a non-bridging O atom of an adjacent chain (H10*A*⋯O51^iii^, 2.64 Å; H52*B*⋯O51^iv^, 2.58 Å; see Table 3 ▸ for symmetry operators). A C—H⋯π type contact is also observed between one H of the other non-O-bridged oxazoline ring and a carbon of an aromatic ring on a parallel chain (H24*B*⋯C20^v^, 2.82 Å; see Table 3 ▸ for symmetry operator). Similarly, the structure of **3** shows two types of close contacts between mol­ecules. One type is between a methyl hydrogen on an oxazoline ring and an oxazoline O atom of an adjacent mol­ecule (H28*A*⋯O9^ii^, 2.55 Å; see Table 4 ▸ for symmetry operator). Structure **3** also shows a close C—H⋯π contacts between the H atom of a CH_2_ group in one oxazoline ring with the aromatic ring of an adjacent mol­ecule (H38*A*⋯C17^i^, 3.01 Å; H38*A*⋯C18^i^, 2.58 Å; see Table 4 ▸ for symmetry operator), resulting in a zigzag chain of Yb(H-**L1**)_3_ units in the solid state (Fig. 7 ▸).

## Database survey   

There are 68 structure in the CSD (version 5.39, update of November, 2018; Groom *et al.*, 2016 ▸) containing a substituted anilido-oxazoline ligand (*R*-**L1** ^−^) coordinated to a transition or main group metal. [COZFIH (Coeffard *et al.*, 2009 ▸); DEJHIK (Cabeza *et al.*, 2006 ▸); EDEBOG (Niwa & Nakada, 2012 ▸); EFICON (Bian *et al.*, 2014 ▸); FONYAI (Mikami *et al.*, 1999 ▸); GIWYES (Chen *et al.*, 2014 ▸); GUTTOF (Inagaki *et al.*, 2010 ▸); ISEWAG (Abbina *et al.*, 2016 ▸); LUNGOS (Bauer *et al.*, 2015*a* ▸); LUNHAF (Bauer *et al.*, 2015*a* ▸); MALVUS, MALWAZ and MALWED (Kieltsch *et al.*, 2010 ▸); MICTID, MICTOJ, MICTUP, MICVAX and MICVEB (Lu *et al.*, 2013 ▸); MUQNAN and MUQNER (Wan *et al.*, 2002 ▸); NANFEP, NANFIT, NANFOZ, NANFUF, NANGAM, NANGEQ, NANGIU and NANGOA (Peng & Chen, 2011 ▸); OCIHOX, OCIHUD and OCIJAL (Cabaleiro *et al.*, 2001 ▸); PUDKUV, PUDLAC, PUDLEG and PUDLIK (Chen *et al.*, 2009*a* ▸); QIFFES and QIFFIW (Abbina & Du, 2012 ▸); RAKTAA (McKeon *et al.*, 2011 ▸); RAMFIW, RAMFOC, RAMFUI, RAMGAP and RAMGET (Chen & Chen, 2011 ▸); RAMKEY (Huang *et al.*, 2017 ▸); ROGWAM (Nakada & Inoue, 2007 ▸); SELVIQ (Nixon & Ward, 2012 ▸); SUYQOS, SUYQUY, SUYRAF and SUYREJ (Castro *et al.*, 2001 ▸); TIMLIL and TIMLOR (Chen *et al.*, 2007 ▸); VOZZOB, VOZZUH and VUBBAX (Bauer *et al.*, 2015*b* ▸); VUQZAK (O’Reilly *et al.*, 2015 ▸); WUGQOF, WUGQUL, WUGRAS and WUGREW (Chen *et al.*, 2009*b* ▸); XIGYEU (Wu *et al.*, 2018 ▸); XOQVEG and XOQVIK (He *et al.*, 2014 ▸); XOYVOW, XOYVUC, XOYWAJ, XOYWEN and XOYWIR (Castro *et al.*, 2002 ▸)]. In contrast, there is only one structure of an unsubstituted anilido-oxazoline ligand (H-**L1** ^−^) coordinated to a transition metal (Cabeza *et al.*, 2006 ▸) and there are no structures of this type with a lanthanide metal. There are 10 lanthanide complexes that have been structurally characterized with the related ligands shown in Fig. 1 ▸ *a* and 1*b* as discussed in the *Chemical context*.

## Synthesis and crystallization   

**General.** All solvents were purchased from Sigma–Aldrich Chemicals and dried by distillation from sodium under nitro­gen. 2-(4′,4′-Dimethyl-2′-oxazolin­yl)aniline was prepared according to Gossage (2009 ▸) and purified by recrystallization from hot toluene. Yb[N(SiMe)_3_)_2_]_3_ was prepared by analogy to the procedure of Bradley *et al.* (1973 ▸) using NaN(SiMe_3_)_2_ and YbCl_3_ and was recrystallized from a hot mixture of hexane and toluene. NMR spectra were recorded on a Bruker AV III 300 MHz Spectrometer in sealable Teflon-valved tubes and were referenced to residual solvent resonances. The line widths at half maximum (ν_1/2_ in Hz) were measured for all paramagnetic resonances in **3** and are reported below. Elemental analyses were performed by Canadian Microanalytical Ltd.

**Synthesis of** [Na_4_(**L1**)_4_]_*n*_. One equivalent of Na[N(SiMe_3_)_2_] (0.183 g, 1.00 mmol) was dissolved in toluene (10 mL) and to this was added 1 equivalent of 2-(4′,4′-dimethyl-2′-oxazolin­yl)aniline (H_2_-**L1**, 0.190 g, 1.00 mmol) in 20 mL toluene under vigorous stirring. The colourless reaction mixture was stirred overnight, filtered through Celite on a sintered glass frit and the solvent removed under reduced pressure to leave a tacky white solid. Recrystallization of the product from a hot mixture of toluene and hexane afforded clear pale-yellow crystals of [Na_4_(**L1**)_4_]_*n*_ (**2**). Yield: 0.178 g (84%). ^1^H NMR (THF-*d*_8_, 300 MHz, 296 K): δ 7.568 (1H, *d*, 3-ar­yl*H*), 7.067 (1H, *t*, 5-ar­yl*H*), 6.635 (1H, *d*, 6-ar­yl*H*), 6.62 (1H, *br s*, N*H*, overlaps previous resonance), 6.459 (1H, *t*, 4-ar­yl*H*), 3.954 (2H, *s*, OC*H_2_*), 1.315 (6H, *s*, C(C*H_3_*)_2_); ^13^C{^1^H} (THF-*d*_8_, 75 MHz, 296 K): δ 163.12 (*C*=N), 150.79 (ar­yl*C*NH), 132.31 (5-ar­yl*C*H), 130.04 (3-ar­yl*C*H), 115.90 (6-ar­yl*C*H), 115.15 (4-ar­yl*C*H), 109.27 (2-ar­yl*C*—C=N), 77.82 (O*C*H_2_), 68.58 [N*C*(CH_3_)_2_], 28.99 [NC(*C*H_3_)_2_].

**Synthesis of** Yb(H-**L1**)_3_ (**3**) **Method A**: A solution of [Na_4_(**L1**)_4_]_*n*_ (**2**) (0.250 g, 0.295 mmol) in THF (10 mL) was added to a suspension of YbCl_3_ (0.062 g, 0.22 mmol) in THF (5 mL) under vigorous stirring. The suspension was stirred overnight at room temperature, filtered through Celite on a sintered glass frit and the filtrate was evaporated to dryness under reduced pressure. The yellow solid was recrystallized from a mixture of toluene and hexane at 243 K overnight. Yield: 0.102 g (63%). **Method B**: A solution of 2-(4′,4′-dimethyl-2′-oxazolin­yl)aniline (0.250 g, 1.31 mmol) in 25 mL toluene was prepared in the glovebox and added by Pasteur pipette to a vigorously stirred solution of Yb[N(SiMe)_3_)_2_]_3_ (0.287 g, 0.438 mmol) in 15 mL of toluene. The pale-yellow solution darkened to golden yellow on stirring overnight. The solution was filtered through Celite on a sintered glass frit and the filtrate was evaporated to dryness under reduced pressure. The orange–yellow solid was recrystallized from a mixture of toluene and hexane at 243 K yielding yellow crystals. Yield: 0.301 g (93%). ^1^H NMR (C_6_D_6_, 300 MHz, 296 K): δ 88.4 (6H, ν_1/2_ = 700 Hz), 49.4 (3H, overlaps next resonance), 47.9 (6H, ν_1/2_ = 350 Hz, overlaps previous resonance), 12.86 (2H, ν_1/2_ = 9 Hz), 11.70 (4H, ν_1/2_ = 12 Hz), 10.93 (4H, ν_1/2_ = 12 Hz), 10.00 (4H, ν_1/2_ = 25 Hz), 9.30 (4H, ν_1/2_ = 70 Hz), 1.26 (2H, t), 0.96 (2H, t), −2.77 (3H, ν_1/2_ = 100 Hz), −3.89 (2H, ν_1/2_ = 14 Hz), −5.38 (2H, ν_1/2_ = 20 Hz), −11.2 (6H, ν_1/2_ ∼150 Hz, overlaps next resonance), −11.4 (6H, ν_1/2_ ∼300 Hz, overlaps previous resonance), −16.0 (3H, ν_1/2_ = 140 Hz), −24.4 (3H, ν_1/2_ = 800 Hz), −77.2 (3H, ν_1/2_ = 600 Hz). Analysis calculated for C_33_H_39_N_6_O_3_Yb (%): C, 53.49; H, 5.31; N, 11.35. Found: C, 53.39; H, 5.22; N, 11.11.

## Refinement   

Crystal data, data collection and structure refinement details are summarized in Table 4 ▸. In Na4(H-**L1**)_4_ (**2**), the H atoms on N1, N15, N29 and N43 were located in a difference map and refined with distance restraints of 0.88 (1) Å. *U*_iso_(H) were freely refined. In Yb(H-**L1**)_3_ (**3**), the H atoms on N1, N15 were added geometrically and refined with distance restraints of 0.88 (1) Å, with *U*_iso_(H) = 1.2*U*_eq_(N). H29 was located in the difference map for geometrical considerations and refined with coordinates riding on N29 with *U*_iso_(H) = 1.2*U*_eq_(N). All the H atoms bonded to carbon were refined in geometrically calculated positions, with C—H= 0.95 (methine), 0.99 (methyl­ene), and 0.98 Å (meth­yl), and with *U*_iso_(H) = 1.2*U*_eq_(C) (methine and methyl­ene) or 1.5*U*_eq_(C) (meth­yl).

## Supplementary Material

Crystal structure: contains datablock(s) Na4H-L142, YbH-L133, global. DOI: 10.1107/S2056989020005034/zl2777sup1.cif

Structure factors: contains datablock(s) Na4H-L142. DOI: 10.1107/S2056989020005034/zl2777Na4H-L142sup2.hkl

Supporting information file. DOI: 10.1107/S2056989020005034/zl2777Na4H-L142sup5.mol

Structure factors: contains datablock(s) YbH-L133. DOI: 10.1107/S2056989020005034/zl2777YbH-L133sup3.hkl

Supporting information file. DOI: 10.1107/S2056989020005034/zl2777YbH-L133sup6.mol

CCDC references: 1996037, 1996036

Additional supporting information:  crystallographic information; 3D view; checkCIF report

## Figures and Tables

**Table 1:** Selected geometric parameters (Å, °) for Na_4_(H-**L1**)_4_ [Chem scheme1]

Na1—O9^i^	2.4003 (15)	Na3—O37^ii^	2.4099 (14)
Na1—N1	2.3465 (18)	Na3—N15	2.3360 (18)
Na1—N12	2.9532 (18)	Na3—N26	2.5515 (16)
Na1—N43	2.3432 (17)	Na3—N29	2.3611 (17)
Na1—N54	2.7348 (17)	Na3—N40	3.0327 (18)
Na2—N1	2.3619 (18)	Na4—N29	2.3701 (18)
Na2—N12	2.3626 (17)	Na4—N40	2.3396 (17)
Na2—N15	2.3411 (18)	Na4—N43	2.3519 (18)
Na2—N26	2.4407 (17)	Na4—N54	2.4035 (17)
			
O9^i^—Na1—N12	107.61 (5)	O37^ii^—Na3—N26	107.75 (5)
O9^i^—Na1—N54	103.04 (5)	O37^ii^—Na3—N40	104.03 (5)
N1—Na1—O9^i^	105.11 (6)	N15—Na3—O37^ii^	141.85 (6)
N1—Na1—N12	65.92 (5)	N15—Na3—N26	72.56 (5)
N1—Na1—N54	92.92 (6)	N15—Na3—N29	108.71 (6)
N43—Na1—O9^i^	142.94 (6)	N15—Na3—N40	85.90 (5)
N43—Na1—N1	111.31 (6)	N26—Na3—N40	147.75 (5)
N43—Na1—N12	93.51 (6)	N29—Na3—O37^ii^	108.68 (6)
N43—Na1—N54	69.08 (5)	N29—Na3—N26	100.25 (6)
N54—Na1—N12	146.27 (5)	N29—Na3—N40	63.74 (5)
N1—Na2—N12	76.60 (6)	N29—Na4—N54	119.12 (6)
N1—Na2—N26	125.43 (6)	N40—Na4—N29	76.15 (6)
N12—Na2—N26	135.48 (6)	N40—Na4—N43	113.77 (6)
N15—Na2—N1	138.81 (6)	N40—Na4—N54	121.58 (6)
N15—Na2—N12	116.21 (7)	N43—Na4—N29	156.58 (6)
N15—Na2—N26	74.56 (6)	N43—Na4—N54	75.06 (6)

Symmetry codes: (i) 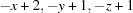; (ii) 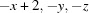.

**Table 2:** Selected geometric parameters (Å, °) for Yb(H-**L1**)_3_ [Chem scheme1]

N1—Yb1	2.252 (3)	N26—Yb1	2.376 (3)
N12—Yb1	2.468 (3)	N29—Yb1	2.234 (3)
N15—Yb1	2.260 (3)	N40—Yb1	2.390 (3)
			
N1—Yb1—N12	74.72 (11)	N26—Yb1—N40	153.87 (10)
N1—Yb1—N15	167.59 (12)	N29—Yb1—N1	89.28 (12)
N1—Yb1—N26	109.02 (11)	N29—Yb1—N12	159.71 (11)
N1—Yb1—N40	86.61 (11)	N29—Yb1—N15	102.04 (12)
N15—Yb1—N12	95.23 (11)	N29—Yb1—N26	82.94 (11)
N15—Yb1—N26	77.79 (11)	N29—Yb1—N40	76.28 (11)
N15—Yb1—N40	91.10 (11)	N40—Yb1—N12	114.28 (10)
N26—Yb1—N12	90.47 (10)		
			
C2—C7—C8—N12	8.6 (7)	C30—C35—C36—N40	−9.3 (6)
C16—C21—C22—N26	8.4 (6)		

**Table 3:** Significant inter­molecular inter­actions (Å) in (2) and (3)

Compound	*D*—H⋯*A*	H⋯*A*	*D*⋯*A*	*D*—H⋯*A*
Na_4_(H-**L1**)_4_ (**2**)	C10—H10*A*⋯O51^iii^	2.64	3.413 (2)	135.3
	C52—H52*B*⋯O51^iv^	2.58	3.439 (2)	145.2
	C24—H24*B*⋯C20^v^	2.82	3.720 (3)	151.4
				
Yb(H-**L1**)_3_ (**3**)	C28—H28*A*⋯O9^ii^	2.55	3.382 (5)	142.8
	C38—H38*A*⋯C17^i^	3.01	3.501 (6)	149.0
	C38—H38*A*⋯C18^i^	2.58	3.548 (6)	165.8

Symmetry codes: (i) −*x* + 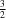, *y* + 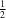, −*z* + 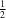; (ii) *x*, *y* − 1, *z*; (iii) *x*, *y* + 1, *z*; (iv) −*x* + 2, −*y*, −*z* + 1; (v) −*x* + 2, −*y* + 1, −*z*.

**Table 4:** Experimental details

	Na_4_(H-**L1**)_4_	Yb(H-**L1**)_3_
Crystal data		
Chemical formula	[Na_4_(C_11_H_13_N_2_O)_4_]	[Yb(C_11_H_13_N_2_O)_3_]
*M*_r_	848.89	740.74
Crystal system, space group	Triclinic, *P* 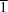	Monoclinic, *P*2_1_/*n*
Temperature (K)	87	86
*a*, *b*, *c* (Å)	10.9545 (5), 11.8785 (5), 18.8415 (8)	10.9428 (5), 9.8253 (5), 28.6089 (14)
α, β, γ (°)	105.266 (1), 97.446 (1), 106.120 (1)	90, 94.722 (1), 90
*V* (Å^3^)	2217.20 (17)	3065.5 (3)
*Z*	2	4
Radiation type	Mo *K*α	Mo *K*α
μ (mm^−1^)	0.12	3.10
Crystal size (mm)	0.26 × 0.18 × 0.15	0.22 × 0.18 × 0.05

Data collection		
Diffractometer	SMART APEX CCD area detector	SMART APEX CCD area detector
Absorption correction	Multi-scan (*SADABS*; Bruker, 2016 ▸)	Multi-scan (*SADABS*; Bruker, 2016 ▸)
*T*_min_, *T*_max_	0.776, 0.983	0.219, 0.262
No. of measured, independent and observed [*I* > 2σ(*I*)] reflections	33551, 12487, 8858	39651, 7061, 5882
*R*_int_	0.054	0.059
(sin θ/λ)_max_ (Å^−1^)	0.704	0.651

Refinement		
*R*[*F* ^2^ > 2σ(*F* ^2^)], *wR*(*F* ^2^), *S*	0.066, 0.158, 1.05	0.032, 0.073, 1.07
No. of reflections	12487	7061
No. of parameters	565	394
No. of restraints	4	0
H-atom treatment	H atoms treated by a mixture of independent and constrained refinement	H-atom parameters constrained
Δρ_max_, Δρ_min_ (e Å^−3^)	0.49, −0.31	0.83, −1.19

Computer programs: *SMART* and *SAINT* (Bruker, 2003 ▸), *SHELXS* (Sheldrick, 2008 ▸), *SHELXL* (Sheldrick, 2015 ▸) and *OLEX2* (Dolomanov *et al.*, 2009 ▸).

## supplementary crystallographic information

### Poly[bis[µ3-2-(4,4-dimethyl-2-oxazolin-2-yl)anilinido][µ2-2-(4,4-dimethyl-2-oxazolin-2-yl)aniline]tetrasodium(I)] (Na4H-L142) Crystal data

**Table:**

[Na_4_(C_11_H_13_N_2_O)_4_]	*Z* = 2
*M**_r_* = 848.89	*F*(000) = 896
Triclinic, *P*1	*D*_x_ = 1.272 Mg m^−^^3^
*a* = 10.9545 (5) Å	Mo *K*α radiation, λ = 0.71073 Å
*b* = 11.8785 (5) Å	Cell parameters from 9632 reflections
*c* = 18.8415 (8) Å	θ = 2.2–29.9°
α = 105.266 (1)°	µ = 0.12 mm^−^^1^
β = 97.446 (1)°	*T* = 87 K
γ = 106.120 (1)°	Needle, pale yellow
*V* = 2217.20 (17) Å^3^	0.26 × 0.18 × 0.15 mm

### Poly[bis[µ3-2-(4,4-dimethyl-2-oxazolin-2-yl)anilinido][µ2-2-(4,4-dimethyl-2-oxazolin-2-yl)aniline]tetrasodium(I)] (Na4H-L142) Data collection

**Table:**

SMART APEX CCD area detector diffractometer	12487 independent reflections
Radiation source: sealed X-ray tube	8858 reflections with *I* > 2σ(*I*)
Graphite monochromator	*R*_int_ = 0.054
Detector resolution: 8.3 pixels mm^-1^	θ_max_ = 30.1°, θ_min_ = 1.9°
φ and ω scans	*h* = −15→14
Absorption correction: multi-scan (SADABS; Bruker, 2016)	*k* = −16→16
*T*_min_ = 0.776, *T*_max_ = 0.983	*l* = −26→26
33551 measured reflections	

### Poly[bis[µ3-2-(4,4-dimethyl-2-oxazolin-2-yl)anilinido][µ2-2-(4,4-dimethyl-2-oxazolin-2-yl)aniline]tetrasodium(I)] (Na4H-L142) Refinement

**Table:**

Refinement on *F*^2^	Primary atom site location: structure-invariant direct methods
Least-squares matrix: full	Secondary atom site location: difference Fourier map
*R*[*F*^2^ > 2σ(*F*^2^)] = 0.066	Hydrogen site location: mixed
*wR*(*F*^2^) = 0.158	H atoms treated by a mixture of independent and constrained refinement
*S* = 1.05	*w* = 1/[σ^2^(*F*_o_^2^) + (0.0734*P*)^2^ + 0.1127*P*] where *P* = (*F*_o_^2^ + 2*F*_c_^2^)/3
12487 reflections	(Δ/σ)_max_ < 0.001
565 parameters	Δρ_max_ = 0.49 e Å^−^^3^
4 restraints	Δρ_min_ = −0.31 e Å^−^^3^

### Poly[bis[µ3-2-(4,4-dimethyl-2-oxazolin-2-yl)anilinido][µ2-2-(4,4-dimethyl-2-oxazolin-2-yl)aniline]tetrasodium(I)] (Na4H-L142) Special details

**Table:**

Experimental. The data collection nominally covered a full sphere of reciprocal space by a combination of 5 sets of ω scans each set at different φ and/or 2θ angles and each scan (10 s exposure) covering -0.300° degrees in ω. The crystal to detector distance was 5.0 cm.
Geometry. All esds (except the esd in the dihedral angle between two l.s. planes) are estimated using the full covariance matrix. The cell esds are taken into account individually in the estimation of esds in distances, angles and torsion angles; correlations between esds in cell parameters are only used when they are defined by crystal symmetry. An approximate (isotropic) treatment of cell esds is used for estimating esds involving l.s. planes.
Refinement. Donor N-H hydrogen atoms located on the difference map and refined with restraints (DFIX).

### Poly[bis[µ3-2-(4,4-dimethyl-2-oxazolin-2-yl)anilinido][µ2-2-(4,4-dimethyl-2-oxazolin-2-yl)aniline]tetrasodium(I)] (Na4H-L142) Fractional atomic coordinates and isotropic or equivalent isotropic displacement parameters (Å^2^)

**Table:**

	*x*	*y*	*z*	*U*_iso_*/*U*_eq_	
Na1	0.95553 (8)	0.27949 (7)	0.39918 (4)	0.02273 (18)	
Na2	1.00695 (7)	0.40238 (7)	0.25656 (4)	0.01977 (17)	
Na3	1.01274 (8)	0.22441 (7)	0.09593 (4)	0.01954 (17)	
Na4	0.96731 (7)	0.11951 (7)	0.24031 (4)	0.01974 (17)	
O9	0.97838 (14)	0.66218 (12)	0.46616 (7)	0.0223 (3)	
O23	1.05206 (13)	0.61390 (12)	0.11145 (8)	0.0240 (3)	
O37	0.92170 (12)	−0.17535 (11)	0.02263 (7)	0.0172 (3)	
O51	0.93031 (13)	−0.09390 (12)	0.40245 (7)	0.0190 (3)	
N1	1.13690 (16)	0.38393 (14)	0.36039 (9)	0.0194 (3)	
H1	1.1910 (17)	0.3417 (18)	0.3515 (12)	0.023 (6)*	
N12	0.93307 (16)	0.49954 (15)	0.36081 (9)	0.0210 (3)	
N15	0.84608 (16)	0.27468 (14)	0.14907 (9)	0.0188 (3)	
H15	0.7835 (15)	0.2151 (14)	0.1541 (11)	0.016 (5)*	
N26	1.08503 (15)	0.45903 (13)	0.15111 (8)	0.0154 (3)	
N29	1.14745 (16)	0.17884 (14)	0.18512 (9)	0.0187 (3)	
H29	1.2125 (16)	0.2441 (15)	0.2135 (11)	0.032 (6)*	
N40	0.90467 (15)	−0.02419 (14)	0.11936 (9)	0.0188 (3)	
N43	0.79137 (16)	0.14194 (14)	0.29563 (9)	0.0177 (3)	
H43	0.7403 (18)	0.1721 (19)	0.2724 (11)	0.025 (6)*	
N54	0.99785 (15)	0.05812 (14)	0.35133 (8)	0.0157 (3)	
C2	1.21029 (19)	0.49537 (17)	0.40943 (10)	0.0185 (4)	
C3	1.3480 (2)	0.52674 (19)	0.43478 (11)	0.0240 (4)	
H3	1.387659	0.465981	0.417427	0.029*	
C4	1.4255 (2)	0.6402 (2)	0.48287 (12)	0.0294 (5)	
H4	1.516605	0.656142	0.497231	0.035*	
C5	1.3727 (2)	0.7324 (2)	0.51099 (11)	0.0307 (5)	
H5	1.426790	0.811193	0.543908	0.037*	
C6	1.2407 (2)	0.70662 (18)	0.48998 (11)	0.0244 (4)	
H6	1.204232	0.768938	0.509515	0.029*	
C7	1.1568 (2)	0.59127 (17)	0.44049 (10)	0.0192 (4)	
C8	1.01968 (19)	0.57690 (16)	0.41890 (10)	0.0179 (4)	
C10	0.8393 (2)	0.62951 (19)	0.43632 (11)	0.0235 (4)	
H10A	0.816298	0.703301	0.433271	0.028*	
H10B	0.787910	0.591249	0.468432	0.028*	
C11	0.8141 (2)	0.53719 (18)	0.35752 (11)	0.0210 (4)	
C13	0.6923 (2)	0.4263 (2)	0.34069 (13)	0.0300 (5)	
H13A	0.682024	0.369426	0.290390	0.045*	
H13B	0.616121	0.454057	0.342445	0.045*	
H13C	0.700305	0.384032	0.378366	0.045*	
C14	0.8081 (2)	0.59894 (19)	0.29623 (11)	0.0285 (5)	
H14A	0.886600	0.670811	0.308121	0.043*	
H14B	0.731041	0.625564	0.293703	0.043*	
H14C	0.802784	0.540086	0.247512	0.043*	
C16	0.78884 (18)	0.34087 (16)	0.11669 (10)	0.0159 (4)	
C17	0.64991 (19)	0.30802 (17)	0.09628 (11)	0.0214 (4)	
H17	0.598324	0.236507	0.105205	0.026*	
C18	0.5880 (2)	0.37338 (19)	0.06493 (12)	0.0256 (4)	
H18	0.495521	0.347610	0.053331	0.031*	
C19	0.6594 (2)	0.4787 (2)	0.04951 (13)	0.0289 (5)	
H19	0.616576	0.524673	0.027426	0.035*	
C20	0.7927 (2)	0.51362 (19)	0.06716 (12)	0.0251 (4)	
H20	0.841448	0.584382	0.056354	0.030*	
C21	0.86026 (18)	0.44926 (17)	0.10054 (10)	0.0169 (4)	
C22	1.00186 (19)	0.50168 (16)	0.12171 (10)	0.0165 (4)	
C24	1.19172 (19)	0.64358 (18)	0.12865 (12)	0.0226 (4)	
H24A	1.234644	0.730969	0.159843	0.027*	
H24B	1.225242	0.628660	0.081893	0.027*	
C25	1.21513 (18)	0.55680 (17)	0.17229 (11)	0.0191 (4)	
C27	1.2462 (2)	0.6203 (2)	0.25726 (12)	0.0300 (5)	
H27A	1.176504	0.652862	0.270847	0.045*	
H27B	1.328909	0.688172	0.271962	0.045*	
H27C	1.253007	0.560475	0.283555	0.045*	
C28	1.3196 (2)	0.5025 (2)	0.14974 (13)	0.0284 (5)	
H28A	1.325909	0.442906	0.176312	0.043*	
H28B	1.403489	0.568677	0.163127	0.043*	
H28C	1.297027	0.460680	0.095216	0.043*	
C30	1.20008 (18)	0.09568 (17)	0.15045 (10)	0.0157 (4)	
C31	1.33777 (19)	0.12026 (18)	0.16195 (11)	0.0215 (4)	
H31	1.393351	0.198458	0.194834	0.026*	
C32	1.3932 (2)	0.03672 (19)	0.12792 (12)	0.0253 (4)	
H32	1.485371	0.058376	0.137784	0.030*	
C33	1.3172 (2)	−0.08002 (19)	0.07886 (12)	0.0254 (4)	
H33	1.356161	−0.138164	0.055791	0.031*	
C34	1.18429 (19)	−0.10787 (18)	0.06512 (11)	0.0200 (4)	
H34	1.131534	−0.186644	0.031580	0.024*	
C35	1.12306 (18)	−0.02447 (16)	0.09871 (10)	0.0153 (4)	
C36	0.98178 (18)	−0.06778 (16)	0.08327 (10)	0.0148 (4)	
C38	0.78300 (18)	−0.19763 (17)	0.01536 (10)	0.0176 (4)	
H38A	0.736096	−0.285692	0.007473	0.021*	
H38B	0.747054	−0.173973	−0.027352	0.021*	
C39	0.77194 (18)	−0.11577 (17)	0.09051 (11)	0.0192 (4)	
C41	0.7463 (2)	−0.1871 (2)	0.14685 (12)	0.0301 (5)	
H41A	0.810457	−0.229695	0.150809	0.045*	
H41B	0.658504	−0.247779	0.129470	0.045*	
H41C	0.753363	−0.129417	0.196360	0.045*	
C42	0.6701 (2)	−0.0533 (2)	0.08068 (13)	0.0298 (5)	
H42A	0.667093	−0.001814	0.130065	0.045*	
H42B	0.584663	−0.116216	0.057219	0.045*	
H42C	0.692872	−0.001556	0.048415	0.045*	
C44	0.71576 (18)	0.05709 (16)	0.32088 (10)	0.0165 (4)	
C45	0.57763 (19)	0.03168 (18)	0.31010 (11)	0.0233 (4)	
H45	0.538590	0.074141	0.282748	0.028*	
C46	0.4998 (2)	−0.05047 (19)	0.33726 (13)	0.0285 (5)	
H46	0.408827	−0.063596	0.328368	0.034*	
C47	0.5513 (2)	−0.11577 (19)	0.37792 (12)	0.0274 (5)	
H47	0.496904	−0.171305	0.397908	0.033*	
C48	0.68180 (19)	−0.09766 (17)	0.38817 (11)	0.0210 (4)	
H48	0.717283	−0.142442	0.415335	0.025*	
C49	0.76637 (18)	−0.01501 (16)	0.36004 (10)	0.0157 (4)	
C50	0.90186 (18)	−0.01006 (16)	0.37005 (9)	0.0149 (4)	
C52	1.07034 (19)	−0.06290 (19)	0.41603 (11)	0.0210 (4)	
H52A	1.095885	−0.138279	0.404131	0.025*	
H52B	1.111240	−0.012808	0.469199	0.025*	
C53	1.10936 (18)	0.01097 (17)	0.36303 (10)	0.0172 (4)	
C55	1.1135 (2)	−0.07327 (18)	0.28694 (11)	0.0220 (4)	
H55A	1.030049	−0.140126	0.266136	0.033*	
H55B	1.183594	−0.108335	0.293956	0.033*	
H55C	1.129591	−0.025254	0.252043	0.033*	
C56	1.23701 (19)	0.11658 (18)	0.39697 (12)	0.0262 (5)	
H56A	1.260152	0.157776	0.359531	0.039*	
H56B	1.305850	0.084140	0.411950	0.039*	
H56C	1.227343	0.175941	0.441303	0.039*	

### Poly[bis[µ3-2-(4,4-dimethyl-2-oxazolin-2-yl)anilinido][µ2-2-(4,4-dimethyl-2-oxazolin-2-yl)aniline]tetrasodium(I)] (Na4H-L142) Atomic displacement parameters (Å^2^)

**Table:**

	*U*^11^	*U*^22^	*U*^33^	*U*^12^	*U*^13^	*U*^23^
Na1	0.0299 (4)	0.0196 (4)	0.0135 (4)	0.0026 (3)	0.0046 (3)	0.0024 (3)
Na2	0.0246 (4)	0.0207 (4)	0.0128 (4)	0.0069 (3)	0.0036 (3)	0.0040 (3)
Na3	0.0284 (4)	0.0190 (4)	0.0132 (4)	0.0111 (3)	0.0049 (3)	0.0044 (3)
Na4	0.0248 (4)	0.0210 (4)	0.0134 (4)	0.0075 (3)	0.0063 (3)	0.0044 (3)
O9	0.0364 (8)	0.0185 (7)	0.0140 (6)	0.0137 (6)	0.0065 (6)	0.0028 (5)
O23	0.0216 (7)	0.0206 (7)	0.0333 (8)	0.0055 (6)	0.0045 (6)	0.0163 (6)
O37	0.0198 (7)	0.0163 (6)	0.0137 (6)	0.0057 (5)	0.0036 (5)	0.0019 (5)
O51	0.0212 (7)	0.0197 (6)	0.0201 (7)	0.0083 (6)	0.0038 (5)	0.0111 (5)
N1	0.0244 (9)	0.0151 (7)	0.0181 (8)	0.0072 (7)	0.0044 (7)	0.0037 (6)
N12	0.0238 (9)	0.0190 (8)	0.0191 (8)	0.0081 (7)	0.0062 (7)	0.0025 (6)
N15	0.0197 (8)	0.0137 (7)	0.0219 (8)	0.0022 (6)	0.0036 (7)	0.0076 (6)
N26	0.0159 (8)	0.0129 (7)	0.0157 (7)	0.0030 (6)	0.0023 (6)	0.0039 (6)
N29	0.0207 (8)	0.0160 (8)	0.0162 (8)	0.0050 (7)	0.0011 (6)	0.0022 (6)
N40	0.0178 (8)	0.0199 (8)	0.0160 (8)	0.0048 (7)	0.0033 (6)	0.0025 (6)
N43	0.0234 (9)	0.0169 (7)	0.0152 (8)	0.0108 (7)	0.0015 (6)	0.0057 (6)
N54	0.0153 (8)	0.0162 (7)	0.0158 (7)	0.0058 (6)	0.0023 (6)	0.0052 (6)
C2	0.0253 (10)	0.0165 (9)	0.0153 (9)	0.0040 (8)	0.0053 (8)	0.0100 (7)
C3	0.0244 (11)	0.0277 (10)	0.0219 (10)	0.0082 (9)	0.0048 (8)	0.0114 (8)
C4	0.0234 (11)	0.0357 (12)	0.0221 (10)	−0.0005 (9)	−0.0001 (9)	0.0108 (9)
C5	0.0346 (12)	0.0255 (11)	0.0178 (10)	−0.0072 (9)	0.0033 (9)	0.0029 (8)
C6	0.0349 (12)	0.0184 (9)	0.0143 (9)	0.0016 (9)	0.0069 (8)	0.0029 (7)
C7	0.0281 (11)	0.0155 (8)	0.0115 (8)	0.0024 (8)	0.0045 (7)	0.0051 (7)
C8	0.0311 (11)	0.0117 (8)	0.0135 (8)	0.0076 (8)	0.0084 (8)	0.0057 (7)
C10	0.0350 (12)	0.0239 (10)	0.0184 (9)	0.0161 (9)	0.0109 (9)	0.0085 (8)
C11	0.0279 (11)	0.0195 (9)	0.0200 (9)	0.0124 (8)	0.0084 (8)	0.0071 (8)
C13	0.0275 (12)	0.0257 (11)	0.0405 (13)	0.0120 (9)	0.0083 (10)	0.0125 (10)
C14	0.0452 (14)	0.0234 (10)	0.0173 (10)	0.0123 (10)	0.0067 (9)	0.0057 (8)
C16	0.0197 (9)	0.0127 (8)	0.0124 (8)	0.0050 (7)	0.0026 (7)	0.0000 (7)
C17	0.0194 (10)	0.0155 (9)	0.0254 (10)	0.0028 (8)	0.0047 (8)	0.0032 (8)
C18	0.0170 (10)	0.0257 (10)	0.0317 (11)	0.0089 (8)	0.0022 (8)	0.0046 (9)
C19	0.0252 (11)	0.0305 (11)	0.0373 (12)	0.0143 (9)	0.0038 (9)	0.0168 (10)
C20	0.0267 (11)	0.0251 (10)	0.0291 (11)	0.0108 (9)	0.0064 (9)	0.0150 (9)
C21	0.0203 (10)	0.0164 (8)	0.0135 (8)	0.0063 (7)	0.0034 (7)	0.0037 (7)
C22	0.0227 (10)	0.0123 (8)	0.0142 (8)	0.0036 (7)	0.0062 (7)	0.0048 (7)
C24	0.0217 (10)	0.0189 (9)	0.0267 (10)	0.0031 (8)	0.0046 (8)	0.0104 (8)
C25	0.0184 (9)	0.0153 (9)	0.0198 (9)	0.0011 (7)	0.0004 (7)	0.0059 (7)
C27	0.0312 (12)	0.0237 (10)	0.0235 (11)	−0.0032 (9)	−0.0030 (9)	0.0058 (9)
C28	0.0193 (10)	0.0258 (10)	0.0429 (13)	0.0077 (9)	0.0075 (9)	0.0147 (10)
C30	0.0188 (9)	0.0173 (8)	0.0132 (8)	0.0051 (7)	0.0029 (7)	0.0095 (7)
C31	0.0181 (10)	0.0207 (9)	0.0227 (10)	0.0023 (8)	0.0009 (8)	0.0080 (8)
C32	0.0165 (10)	0.0299 (11)	0.0329 (11)	0.0081 (8)	0.0057 (8)	0.0145 (9)
C33	0.0244 (11)	0.0261 (10)	0.0318 (11)	0.0137 (9)	0.0102 (9)	0.0111 (9)
C34	0.0228 (10)	0.0194 (9)	0.0183 (9)	0.0077 (8)	0.0044 (8)	0.0059 (7)
C35	0.0172 (9)	0.0178 (9)	0.0131 (8)	0.0065 (7)	0.0036 (7)	0.0074 (7)
C36	0.0214 (9)	0.0135 (8)	0.0105 (8)	0.0063 (7)	0.0023 (7)	0.0054 (7)
C38	0.0168 (9)	0.0178 (9)	0.0156 (9)	0.0035 (7)	0.0009 (7)	0.0045 (7)
C39	0.0177 (9)	0.0172 (9)	0.0185 (9)	0.0032 (7)	0.0046 (7)	0.0015 (7)
C41	0.0381 (13)	0.0269 (11)	0.0210 (10)	0.0044 (10)	0.0120 (9)	0.0045 (9)
C42	0.0164 (10)	0.0243 (10)	0.0412 (13)	0.0054 (8)	0.0034 (9)	0.0007 (9)
C44	0.0197 (9)	0.0156 (8)	0.0106 (8)	0.0066 (7)	0.0021 (7)	−0.0018 (7)
C45	0.0201 (10)	0.0223 (10)	0.0234 (10)	0.0098 (8)	−0.0018 (8)	0.0007 (8)
C46	0.0149 (10)	0.0254 (10)	0.0363 (12)	0.0032 (8)	0.0040 (9)	−0.0007 (9)
C47	0.0217 (11)	0.0253 (10)	0.0290 (11)	−0.0001 (9)	0.0083 (9)	0.0055 (9)
C48	0.0233 (10)	0.0183 (9)	0.0179 (9)	0.0031 (8)	0.0033 (8)	0.0045 (7)
C49	0.0180 (9)	0.0152 (8)	0.0107 (8)	0.0040 (7)	0.0021 (7)	0.0009 (7)
C50	0.0200 (9)	0.0132 (8)	0.0100 (8)	0.0055 (7)	0.0005 (7)	0.0025 (6)
C52	0.0203 (10)	0.0259 (10)	0.0191 (9)	0.0110 (8)	0.0017 (8)	0.0083 (8)
C53	0.0179 (9)	0.0161 (8)	0.0178 (9)	0.0077 (7)	0.0019 (7)	0.0041 (7)
C55	0.0232 (10)	0.0239 (10)	0.0214 (10)	0.0116 (8)	0.0062 (8)	0.0066 (8)
C56	0.0169 (10)	0.0215 (10)	0.0355 (12)	0.0059 (8)	0.0026 (9)	0.0031 (9)

### Poly[bis[µ3-2-(4,4-dimethyl-2-oxazolin-2-yl)anilinido][µ2-2-(4,4-dimethyl-2-oxazolin-2-yl)aniline]tetrasodium(I)] (Na4H-L142) Geometric parameters (Å, º)

**Table:**

Na1—O9^i^	2.4003 (15)	C16—C17	1.434 (3)
Na1—N1	2.3465 (18)	C16—C21	1.439 (3)
Na1—N12	2.9532 (18)	C17—H17	0.9500
Na1—N43	2.3432 (17)	C17—C18	1.357 (3)
Na1—N54	2.7348 (17)	C18—H18	0.9500
Na2—N1	2.3619 (18)	C18—C19	1.403 (3)
Na2—N12	2.3626 (17)	C19—H19	0.9500
Na2—N15	2.3411 (18)	C19—C20	1.370 (3)
Na2—N26	2.4407 (17)	C20—H20	0.9500
Na3—O37^ii^	2.4099 (14)	C20—C21	1.405 (3)
Na3—N15	2.3360 (18)	C21—C22	1.458 (3)
Na3—N26	2.5515 (16)	C24—H24A	0.9900
Na3—N29	2.3611 (17)	C24—H24B	0.9900
Na3—N40	3.0327 (18)	C24—C25	1.531 (3)
Na4—N29	2.3701 (18)	C25—C27	1.526 (3)
Na4—N40	2.3396 (17)	C25—C28	1.516 (3)
Na4—N43	2.3519 (18)	C27—H27A	0.9800
Na4—N54	2.4035 (17)	C27—H27B	0.9800
O9—C8	1.381 (2)	C27—H27C	0.9800
O9—C10	1.457 (3)	C28—H28A	0.9800
O23—C22	1.369 (2)	C28—H28B	0.9800
O23—C24	1.443 (2)	C28—H28C	0.9800
O37—C36	1.393 (2)	C30—C31	1.430 (3)
O37—C38	1.450 (2)	C30—C35	1.444 (2)
O51—C50	1.380 (2)	C31—H31	0.9500
O51—C52	1.445 (2)	C31—C32	1.366 (3)
N1—H1	0.882 (9)	C32—H32	0.9500
N1—C2	1.351 (2)	C32—C33	1.398 (3)
N12—C8	1.288 (2)	C33—H33	0.9500
N12—C11	1.490 (3)	C33—C34	1.373 (3)
N15—H15	0.873 (9)	C34—H34	0.9500
N15—C16	1.346 (2)	C34—C35	1.407 (3)
N26—C22	1.291 (2)	C35—C36	1.452 (3)
N26—C25	1.493 (2)	C38—H38A	0.9900
N29—H29	0.878 (10)	C38—H38B	0.9900
N29—C30	1.345 (2)	C38—C39	1.533 (3)
N40—C36	1.285 (2)	C39—C41	1.530 (3)
N40—C39	1.483 (2)	C39—C42	1.519 (3)
N43—H43	0.873 (9)	C41—H41A	0.9800
N43—C44	1.349 (2)	C41—H41C	0.9800
N54—C50	1.288 (2)	C42—H42A	0.9800
N54—C53	1.493 (2)	C42—H42B	0.9800
C2—C3	1.432 (3)	C42—H42C	0.9800
C2—C7	1.446 (3)	C44—C45	1.434 (3)
C3—H3	0.9500	C44—C49	1.441 (3)
C3—C4	1.371 (3)	C45—H45	0.9500
C4—H4	0.9500	C45—C46	1.362 (3)
C4—C5	1.393 (3)	C46—H46	0.9500
C5—H5	0.9500	C46—C47	1.397 (3)
C5—C6	1.372 (3)	C47—H47	0.9500
C6—H6	0.9500	C47—C48	1.365 (3)
C6—C7	1.413 (3)	C48—H48	0.9500
C7—C8	1.454 (3)	C48—C49	1.414 (3)
C10—H10A	0.9900	C49—C50	1.454 (3)
C10—H10B	0.9900	C52—H52A	0.9900
C10—C11	1.533 (3)	C52—H52B	0.9900
C11—C13	1.519 (3)	C52—C53	1.521 (3)
C11—C14	1.526 (3)	C53—C55	1.532 (3)
C13—H13A	0.9800	C53—C56	1.520 (3)
C13—H13B	0.9800	C55—H55B	0.9800
C13—H13C	0.9800	C55—H55C	0.9800
C14—H14A	0.9800	C56—H56A	0.9800
C14—H14B	0.9800	C56—H56B	0.9800
C14—H14C	0.9800		
			
O9^i^—Na1—N12	107.61 (5)	N15—C16—C21	123.45 (17)
O9^i^—Na1—N54	103.04 (5)	C17—C16—C21	114.92 (17)
N1—Na1—O9^i^	105.11 (6)	C16—C17—H17	118.2
N1—Na1—N12	65.92 (5)	C18—C17—C16	123.67 (18)
N1—Na1—N54	92.92 (6)	C18—C17—H17	118.2
N43—Na1—O9^i^	142.94 (6)	C17—C18—H18	119.7
N43—Na1—N1	111.31 (6)	C17—C18—C19	120.53 (19)
N43—Na1—N12	93.51 (6)	C19—C18—H18	119.7
N43—Na1—N54	69.08 (5)	C18—C19—H19	120.9
N54—Na1—N12	146.27 (5)	C20—C19—C18	118.25 (19)
N1—Na2—N12	76.60 (6)	C20—C19—H19	120.9
N1—Na2—N26	125.43 (6)	C19—C20—H20	118.6
N12—Na2—N26	135.48 (6)	C19—C20—C21	122.87 (19)
N15—Na2—N1	138.81 (6)	C21—C20—H20	118.6
N15—Na2—N12	116.21 (7)	C16—C21—C22	122.91 (16)
N15—Na2—N26	74.56 (6)	C20—C21—C16	119.76 (18)
O37^ii^—Na3—N26	107.75 (5)	C20—C21—C22	117.22 (17)
O37^ii^—Na3—N40	104.03 (5)	O23—C22—C21	114.87 (16)
N15—Na3—O37^ii^	141.85 (6)	N26—C22—O23	116.13 (16)
N15—Na3—N26	72.56 (5)	N26—C22—C21	128.95 (16)
N15—Na3—N29	108.71 (6)	O23—C24—H24A	110.9
N15—Na3—N40	85.90 (5)	O23—C24—H24B	110.9
N26—Na3—N40	147.75 (5)	O23—C24—C25	104.31 (15)
N29—Na3—O37^ii^	108.68 (6)	H24A—C24—H24B	108.9
N29—Na3—N26	100.25 (6)	C25—C24—H24A	110.9
N29—Na3—N40	63.74 (5)	C25—C24—H24B	110.9
N29—Na4—N54	119.12 (6)	N26—C25—C24	102.30 (14)
N40—Na4—N29	76.15 (6)	N26—C25—C27	108.12 (16)
N40—Na4—N43	113.77 (6)	N26—C25—C28	111.12 (15)
N40—Na4—N54	121.58 (6)	C27—C25—C24	111.40 (16)
N43—Na4—N29	156.58 (6)	C28—C25—C24	112.81 (16)
N43—Na4—N54	75.06 (6)	C28—C25—C27	110.72 (17)
C8—O9—Na1^i^	121.72 (11)	C25—C27—H27A	109.5
C8—O9—C10	106.14 (14)	C25—C27—H27B	109.5
C10—O9—Na1^i^	115.61 (11)	C25—C27—H27C	109.5
C22—O23—C24	106.11 (14)	H27A—C27—H27B	109.5
C36—O37—Na3^ii^	125.75 (11)	H27A—C27—H27C	109.5
C36—O37—C38	105.65 (13)	H27B—C27—H27C	109.5
C38—O37—Na3^ii^	114.12 (10)	C25—C28—H28A	109.5
C50—O51—C52	105.58 (14)	C25—C28—H28B	109.5
Na1—N1—Na2	93.03 (7)	C25—C28—H28C	109.5
Na1—N1—H1	113.0 (14)	H28A—C28—H28B	109.5
Na2—N1—H1	118.3 (14)	H28A—C28—H28C	109.5
C2—N1—Na1	115.17 (12)	H28B—C28—H28C	109.5
C2—N1—Na2	111.80 (12)	N29—C30—C31	122.19 (17)
C2—N1—H1	105.6 (14)	N29—C30—C35	123.07 (17)
Na2—N12—Na1	79.12 (5)	C31—C30—C35	114.74 (17)
C8—N12—Na1	94.47 (11)	C30—C31—H31	118.4
C8—N12—Na2	117.61 (13)	C32—C31—C30	123.14 (18)
C8—N12—C11	108.22 (15)	C32—C31—H31	118.4
C11—N12—Na1	124.85 (12)	C31—C32—H32	119.3
C11—N12—Na2	126.23 (12)	C31—C32—C33	121.42 (19)
Na2—N15—H15	117.9 (13)	C33—C32—H32	119.3
Na3—N15—Na2	87.12 (6)	C32—C33—H33	121.1
Na3—N15—H15	118.1 (13)	C34—C33—C32	117.83 (19)
C16—N15—Na2	111.22 (11)	C34—C33—H33	121.1
C16—N15—Na3	115.06 (12)	C33—C34—H34	118.7
C16—N15—H15	106.7 (14)	C33—C34—C35	122.67 (18)
Na2—N26—Na3	80.39 (5)	C35—C34—H34	118.7
C22—N26—Na2	106.64 (12)	C30—C35—C36	122.34 (16)
C22—N26—Na3	110.53 (11)	C34—C35—C30	120.20 (17)
C22—N26—C25	107.27 (15)	C34—C35—C36	117.32 (16)
C25—N26—Na2	114.75 (11)	O37—C36—C35	115.12 (15)
C25—N26—Na3	132.60 (12)	N40—C36—O37	115.49 (16)
Na3—N29—Na4	88.98 (6)	N40—C36—C35	129.31 (16)
Na3—N29—H29	112.6 (15)	O37—C38—H38A	110.9
Na4—N29—H29	115.7 (15)	O37—C38—H38B	110.9
C30—N29—Na3	110.70 (12)	O37—C38—C39	104.18 (14)
C30—N29—Na4	121.06 (12)	H38A—C38—H38B	108.9
C30—N29—H29	106.8 (16)	C39—C38—H38A	110.9
Na4—N40—Na3	74.97 (5)	C39—C38—H38B	110.9
C36—N40—Na3	91.91 (11)	N40—C39—C38	102.51 (14)
C36—N40—Na4	125.10 (13)	N40—C39—C41	107.69 (16)
C36—N40—C39	107.94 (15)	N40—C39—C42	111.29 (16)
C39—N40—Na3	129.29 (11)	C41—C39—C38	111.86 (16)
C39—N40—Na4	121.69 (11)	C42—C39—C38	112.55 (17)
Na1—N43—Na4	83.79 (6)	C42—C39—C41	110.59 (17)
Na1—N43—H43	117.7 (15)	C39—C41—H41A	109.5
Na4—N43—H43	114.4 (15)	C39—C41—H41B	109.5
C44—N43—Na1	106.88 (11)	C39—C41—H41C	109.5
C44—N43—Na4	125.00 (12)	H41A—C41—H41B	109.5
C44—N43—H43	107.5 (15)	H41A—C41—H41C	109.5
Na4—N54—Na1	74.89 (5)	H41B—C41—H41C	109.5
C50—N54—Na1	99.29 (11)	C39—C42—H42A	109.5
C50—N54—Na4	121.60 (12)	C39—C42—H42B	109.5
C50—N54—C53	107.20 (15)	C39—C42—H42C	109.5
C53—N54—Na1	136.32 (11)	H42A—C42—H42B	109.5
C53—N54—Na4	116.25 (11)	H42A—C42—H42C	109.5
N1—C2—C3	121.90 (18)	H42B—C42—H42C	109.5
N1—C2—C7	123.10 (18)	N43—C44—C45	121.95 (17)
C3—C2—C7	115.00 (17)	N43—C44—C49	123.11 (17)
C2—C3—H3	118.4	C45—C44—C49	114.95 (17)
C4—C3—C2	123.2 (2)	C44—C45—H45	118.5
C4—C3—H3	118.4	C46—C45—C44	123.07 (19)
C3—C4—H4	119.5	C46—C45—H45	118.5
C3—C4—C5	121.0 (2)	C45—C46—H46	119.4
C5—C4—H4	119.5	C45—C46—C47	121.23 (19)
C4—C5—H5	120.8	C47—C46—H46	119.4
C6—C5—C4	118.36 (19)	C46—C47—H47	120.9
C6—C5—H5	120.8	C48—C47—C46	118.22 (19)
C5—C6—H6	118.6	C48—C47—H47	120.9
C5—C6—C7	122.8 (2)	C47—C48—H48	118.6
C7—C6—H6	118.6	C47—C48—C49	122.72 (19)
C2—C7—C8	122.39 (16)	C49—C48—H48	118.6
C6—C7—C2	119.62 (19)	C44—C49—C50	122.67 (16)
C6—C7—C8	117.86 (18)	C48—C49—C44	119.72 (17)
O9—C8—C7	114.85 (16)	C48—C49—C50	117.57 (17)
N12—C8—O9	115.72 (17)	O51—C50—C49	114.72 (15)
N12—C8—C7	129.38 (17)	N54—C50—O51	115.49 (16)
O9—C10—H10A	110.8	N54—C50—C49	129.76 (17)
O9—C10—H10B	110.8	O51—C52—H52A	111.0
O9—C10—C11	104.51 (15)	O51—C52—H52B	111.0
H10A—C10—H10B	108.9	O51—C52—C53	103.90 (14)
C11—C10—H10A	110.9	H52A—C52—H52B	109.0
C11—C10—H10B	110.8	C53—C52—H52A	111.0
N12—C11—C10	102.57 (16)	C53—C52—H52B	111.0
N12—C11—C13	110.96 (16)	N54—C53—C52	102.01 (14)
N12—C11—C14	108.21 (16)	N54—C53—C55	108.32 (15)
C13—C11—C10	112.78 (17)	N54—C53—C56	111.15 (15)
C13—C11—C14	110.34 (18)	C52—C53—C55	110.98 (16)
C14—C11—C10	111.67 (16)	C56—C53—C52	112.86 (16)
C11—C13—H13A	109.5	C56—C53—C55	111.09 (16)
C11—C13—H13B	109.5	C53—C55—H55A	109.5
C11—C13—H13C	109.5	C53—C55—H55B	109.5
H13A—C13—H13B	109.5	C53—C55—H55C	109.5
H13A—C13—H13C	109.5	H55A—C55—H55B	109.5
H13B—C13—H13C	109.5	H55A—C55—H55C	109.5
C11—C14—H14A	109.5	H55B—C55—H55C	109.5
C11—C14—H14B	109.5	C53—C56—H56A	109.5
C11—C14—H14C	109.5	C53—C56—H56B	109.5
H14A—C14—H14B	109.5	C53—C56—H56C	109.5
H14A—C14—H14C	109.5	H56A—C56—H56B	109.5
H14B—C14—H14C	109.5	H56A—C56—H56C	109.5
N15—C16—C17	121.62 (17)	H56B—C56—H56C	109.5
			
Na1^i^—O9—C8—N12	139.42 (14)	N43—C44—C45—C46	−177.71 (18)
Na1^i^—O9—C8—C7	−43.15 (19)	N43—C44—C49—C48	176.76 (16)
Na1^i^—O9—C10—C11	−151.40 (11)	N43—C44—C49—C50	−5.4 (3)
Na1—N1—C2—C3	127.34 (16)	C2—C3—C4—C5	0.9 (3)
Na1—N1—C2—C7	−52.4 (2)	C2—C7—C8—O9	164.80 (16)
Na1—N12—C8—O9	−121.99 (13)	C2—C7—C8—N12	−18.2 (3)
Na1—N12—C8—C7	61.0 (2)	C3—C2—C7—C6	2.0 (2)
Na1—N12—C11—C10	94.69 (15)	C3—C2—C7—C8	177.76 (16)
Na1—N12—C11—C13	−26.0 (2)	C3—C4—C5—C6	0.6 (3)
Na1—N12—C11—C14	−147.17 (13)	C4—C5—C6—C7	−0.7 (3)
Na1—N43—C44—C45	125.50 (15)	C5—C6—C7—C2	−0.6 (3)
Na1—N43—C44—C49	−54.82 (19)	C5—C6—C7—C8	−176.61 (18)
Na1—N54—C50—O51	−137.84 (12)	C6—C7—C8—O9	−19.3 (2)
Na1—N54—C50—C49	44.4 (2)	C6—C7—C8—N12	157.67 (19)
Na1—N54—C53—C52	105.62 (16)	C7—C2—C3—C4	−2.1 (3)
Na1—N54—C53—C55	−137.24 (14)	C8—O9—C10—C11	−13.18 (18)
Na1—N54—C53—C56	−14.9 (2)	C8—N12—C11—C10	−14.5 (2)
Na2—N1—C2—C3	−128.14 (16)	C8—N12—C11—C13	−135.21 (18)
Na2—N1—C2—C7	52.2 (2)	C8—N12—C11—C14	103.60 (18)
Na2—N12—C8—O9	157.96 (12)	C10—O9—C8—N12	4.4 (2)
Na2—N12—C8—C7	−19.0 (3)	C10—O9—C8—C7	−178.22 (15)
Na2—N12—C11—C10	−162.31 (12)	C11—N12—C8—O9	7.0 (2)
Na2—N12—C11—C13	77.02 (19)	C11—N12—C8—C7	−169.98 (18)
Na2—N12—C11—C14	−44.2 (2)	C16—C17—C18—C19	0.9 (3)
Na2—N15—C16—C17	−130.62 (15)	C16—C21—C22—O23	−171.28 (16)
Na2—N15—C16—C21	48.8 (2)	C16—C21—C22—N26	5.9 (3)
Na2—N26—C22—O23	128.93 (14)	C17—C16—C21—C20	0.0 (2)
Na2—N26—C22—C21	−48.2 (2)	C17—C16—C21—C22	175.84 (16)
Na2—N26—C25—C24	−133.65 (12)	C17—C18—C19—C20	−0.2 (3)
Na2—N26—C25—C27	−15.98 (19)	C18—C19—C20—C21	−0.6 (3)
Na2—N26—C25—C28	105.72 (15)	C19—C20—C21—C16	0.8 (3)
Na3^ii^—O37—C36—N40	142.02 (13)	C19—C20—C21—C22	−175.37 (19)
Na3^ii^—O37—C36—C35	−40.80 (19)	C20—C21—C22—O23	4.7 (2)
Na3^ii^—O37—C38—C39	−158.32 (11)	C20—C21—C22—N26	−178.11 (19)
Na3—N15—C16—C17	132.37 (15)	C21—C16—C17—C18	−0.8 (3)
Na3—N15—C16—C21	−48.3 (2)	C22—O23—C24—C25	−16.83 (19)
Na3—N26—C22—O23	−145.32 (13)	C22—N26—C25—C24	−15.39 (19)
Na3—N26—C22—C21	37.5 (2)	C22—N26—C25—C27	102.28 (18)
Na3—N26—C25—C24	126.32 (14)	C22—N26—C25—C28	−136.02 (17)
Na3—N26—C25—C27	−116.02 (16)	C24—O23—C22—N26	7.7 (2)
Na3—N26—C25—C28	5.7 (2)	C24—O23—C22—C21	−174.71 (15)
Na3—N29—C30—C31	122.47 (16)	C25—N26—C22—O23	5.5 (2)
Na3—N29—C30—C35	−57.2 (2)	C25—N26—C22—C21	−171.62 (17)
Na3—N40—C36—O37	−124.35 (13)	C30—C31—C32—C33	0.0 (3)
Na3—N40—C36—C35	58.96 (19)	C30—C35—C36—O37	169.03 (15)
Na3—N40—C39—C38	90.53 (16)	C30—C35—C36—N40	−14.3 (3)
Na3—N40—C39—C41	−151.36 (13)	C31—C30—C35—C34	1.2 (2)
Na3—N40—C39—C42	−30.0 (2)	C31—C30—C35—C36	176.71 (16)
Na4—N29—C30—C31	−135.69 (15)	C31—C32—C33—C34	0.8 (3)
Na4—N29—C30—C35	44.7 (2)	C32—C33—C34—C35	−0.5 (3)
Na4—N40—C36—O37	162.73 (11)	C33—C34—C35—C30	−0.5 (3)
Na4—N40—C36—C35	−14.0 (3)	C33—C34—C35—C36	−176.24 (18)
Na4—N40—C39—C38	−173.06 (11)	C34—C35—C36—O37	−15.3 (2)
Na4—N40—C39—C41	−54.96 (19)	C34—C35—C36—N40	161.36 (19)
Na4—N40—C39—C42	66.40 (19)	C35—C30—C31—C32	−1.0 (3)
Na4—N43—C44—C45	−140.23 (15)	C36—O37—C38—C39	−16.11 (17)
Na4—N43—C44—C49	39.4 (2)	C36—N40—C39—C38	−17.54 (19)
Na4—N54—C50—O51	144.26 (12)	C36—N40—C39—C41	100.57 (18)
Na4—N54—C50—C49	−33.5 (2)	C36—N40—C39—C42	−138.07 (17)
Na4—N54—C53—C52	−158.89 (11)	C38—O37—C36—N40	5.6 (2)
Na4—N54—C53—C55	−41.75 (17)	C38—O37—C36—C35	−177.25 (14)
Na4—N54—C53—C56	80.56 (17)	C39—N40—C36—O37	8.2 (2)
O9—C10—C11—N12	16.47 (18)	C39—N40—C36—C35	−168.45 (17)
O9—C10—C11—C13	135.88 (17)	C44—C45—C46—C47	0.0 (3)
O9—C10—C11—C14	−99.20 (19)	C44—C49—C50—O51	−174.02 (15)
O23—C24—C25—N26	19.30 (18)	C44—C49—C50—N54	3.8 (3)
O23—C24—C25—C27	−96.01 (18)	C45—C44—C49—C48	−3.5 (2)
O23—C24—C25—C28	138.75 (16)	C45—C44—C49—C50	174.26 (16)
O37—C38—C39—N40	20.13 (18)	C45—C46—C47—C48	−1.7 (3)
O37—C38—C39—C41	−94.98 (18)	C46—C47—C48—C49	0.6 (3)
O37—C38—C39—C42	139.79 (16)	C47—C48—C49—C44	2.1 (3)
O51—C52—C53—N54	23.61 (17)	C47—C48—C49—C50	−175.82 (18)
O51—C52—C53—C55	−91.59 (18)	C48—C49—C50—O51	3.8 (2)
O51—C52—C53—C56	142.96 (16)	C48—C49—C50—N54	−178.38 (18)
N1—C2—C3—C4	178.15 (18)	C49—C44—C45—C46	2.6 (3)
N1—C2—C7—C6	−178.33 (17)	C50—O51—C52—C53	−20.39 (18)
N1—C2—C7—C8	−2.5 (3)	C50—N54—C53—C52	−19.05 (18)
N15—C16—C17—C18	178.63 (19)	C50—N54—C53—C55	98.09 (17)
N15—C16—C21—C20	−179.45 (17)	C50—N54—C53—C56	−139.59 (17)
N15—C16—C21—C22	−3.6 (3)	C52—O51—C50—N54	9.1 (2)
N29—C30—C31—C32	179.32 (18)	C52—O51—C50—C49	−172.80 (15)
N29—C30—C35—C34	−179.10 (17)	C53—N54—C50—O51	7.0 (2)
N29—C30—C35—C36	−3.6 (3)	C53—N54—C50—C49	−170.76 (17)

Symmetry codes: (i) −*x*+2, −*y*+1, −*z*+1; (ii) −*x*+2, −*y*, −*z*.

### Tris[2-(4,4-dimethyl-2-oxazolin-2-yl)anilinido]ytterbium(III) (YbH-L133) Crystal data

**Table:**

[Yb(C_11_H_13_N_2_O)_3_]	*F*(000) = 1492
*M**_r_* = 740.74	*D*_x_ = 1.605 Mg m^−^^3^
Monoclinic, *P*2_1_/*n*	Mo *K*α radiation, λ = 0.71073 Å
*a* = 10.9428 (5) Å	Cell parameters from 7270 reflections
*b* = 9.8253 (5) Å	θ = 2.2–24.6°
*c* = 28.6089 (14) Å	µ = 3.10 mm^−^^1^
β = 94.722 (1)°	*T* = 86 K
*V* = 3065.5 (3) Å^3^	Plate, yellow
*Z* = 4	0.22 × 0.18 × 0.05 mm

### Tris[2-(4,4-dimethyl-2-oxazolin-2-yl)anilinido]ytterbium(III) (YbH-L133) Data collection

**Table:**

SMART APEX CCD area detector diffractometer	7061 independent reflections
Radiation source: sealed X-ray tube	5882 reflections with *I* > 2σ(*I*)
Graphite monochromator	*R*_int_ = 0.059
Detector resolution: 8.3 pixels mm^-1^	θ_max_ = 27.6°, θ_min_ = 1.9°
φ and ω scans	*h* = −14→14
Absorption correction: multi-scan (SADABS; Bruker, 2016)	*k* = −12→12
*T*_min_ = 0.219, *T*_max_ = 0.262	*l* = −37→37
39651 measured reflections	

### Tris[2-(4,4-dimethyl-2-oxazolin-2-yl)anilinido]ytterbium(III) (YbH-L133) Refinement

**Table:**

Refinement on *F*^2^	Primary atom site location: heavy-atom method
Least-squares matrix: full	Hydrogen site location: mixed
*R*[*F*^2^ > 2σ(*F*^2^)] = 0.032	H-atom parameters constrained
*wR*(*F*^2^) = 0.073	*w* = 1/[σ^2^(*F*_o_^2^) + (0.0248*P*)^2^ + 4.5847*P*] where *P* = (*F*_o_^2^ + 2*F*_c_^2^)/3
*S* = 1.07	(Δ/σ)_max_ = 0.002
7061 reflections	Δρ_max_ = 0.83 e Å^−^^3^
394 parameters	Δρ_min_ = −1.19 e Å^−^^3^
0 restraints	

### Tris[2-(4,4-dimethyl-2-oxazolin-2-yl)anilinido]ytterbium(III) (YbH-L133) Special details

**Table:**

Experimental. The data collection nominally covered a full sphere of reciprocal space by a combination of 5 sets of ω scans each set at different φ and/or 2θ angles and each scan (20 s exposure) covering -0.300° degrees in ω. The crystal to detector distance was 5.0 cm.
Geometry. All esds (except the esd in the dihedral angle between two l.s. planes) are estimated using the full covariance matrix. The cell esds are taken into account individually in the estimation of esds in distances, angles and torsion angles; correlations between esds in cell parameters are only used when they are defined by crystal symmetry. An approximate (isotropic) treatment of cell esds is used for estimating esds involving l.s. planes.

### Tris[2-(4,4-dimethyl-2-oxazolin-2-yl)anilinido]ytterbium(III) (YbH-L133) Fractional atomic coordinates and isotropic or equivalent isotropic displacement parameters (Å^2^)

**Table:**

	*x*	*y*	*z*	*U*_iso_*/*U*_eq_	
C2	1.0818 (3)	0.2384 (4)	0.10704 (13)	0.0200 (8)	
C3	1.2109 (4)	0.2483 (4)	0.11734 (15)	0.0262 (9)	
H3	1.253065	0.173718	0.132394	0.031*	
C4	1.2766 (4)	0.3596 (5)	0.10659 (16)	0.0316 (10)	
H4	1.362244	0.362906	0.115191	0.038*	
C5	1.2190 (4)	0.4686 (5)	0.08310 (17)	0.0340 (11)	
H5	1.265345	0.544940	0.074364	0.041*	
C6	1.0945 (4)	0.4649 (4)	0.07264 (16)	0.0283 (10)	
H6	1.055257	0.539088	0.056247	0.034*	
C7	1.0229 (4)	0.3534 (4)	0.08564 (13)	0.0199 (8)	
C8	0.8911 (4)	0.3647 (4)	0.07860 (14)	0.0240 (9)	
C10	0.7215 (4)	0.4633 (5)	0.0446 (2)	0.0415 (13)	
H10A	0.700654	0.427434	0.012561	0.050*	
H10B	0.679548	0.551716	0.047774	0.050*	
C11	0.6856 (4)	0.3624 (4)	0.08159 (16)	0.0261 (9)	
C13	0.6499 (4)	0.4350 (5)	0.12536 (18)	0.0402 (12)	
H13A	0.717992	0.492735	0.137852	0.060*	
H13B	0.631465	0.367551	0.149013	0.060*	
H13C	0.577374	0.491481	0.117436	0.060*	
C14	0.5842 (4)	0.2703 (4)	0.06114 (15)	0.0267 (9)	
H14A	0.512628	0.325250	0.050405	0.040*	
H14B	0.561617	0.206075	0.085192	0.040*	
H14C	0.612722	0.219744	0.034575	0.040*	
C16	0.5330 (3)	−0.0138 (4)	0.14097 (13)	0.0178 (8)	
C17	0.4277 (3)	−0.0117 (4)	0.16733 (14)	0.0215 (8)	
H17	0.428448	0.045669	0.194088	0.026*	
C18	0.3261 (4)	−0.0887 (4)	0.15562 (15)	0.0244 (9)	
H18	0.258888	−0.085095	0.174572	0.029*	
C19	0.3196 (4)	−0.1728 (4)	0.11627 (15)	0.0270 (9)	
H19	0.248910	−0.226754	0.108288	0.032*	
C20	0.4175 (4)	−0.1757 (4)	0.08933 (14)	0.0238 (9)	
H20	0.413171	−0.232525	0.062388	0.029*	
C21	0.5241 (3)	−0.0979 (4)	0.09994 (13)	0.0178 (8)	
C22	0.6183 (4)	−0.0995 (4)	0.06692 (13)	0.0195 (8)	
C24	0.6760 (4)	−0.1389 (5)	−0.00564 (14)	0.0298 (10)	
H24A	0.643468	−0.071623	−0.029244	0.036*	
H24B	0.700649	−0.221940	−0.022058	0.036*	
C25	0.7847 (3)	−0.0797 (4)	0.02494 (13)	0.0211 (8)	
C27	0.8359 (4)	0.0464 (4)	0.00316 (14)	0.0243 (9)	
H27A	0.870264	0.021891	−0.026275	0.036*	
H27B	0.900484	0.085664	0.024844	0.036*	
H27C	0.770182	0.113260	−0.003178	0.036*	
C28	0.8841 (4)	−0.1848 (4)	0.03572 (15)	0.0270 (9)	
H28A	0.849832	−0.263196	0.051309	0.041*	
H28B	0.950454	−0.144768	0.056380	0.041*	
H28C	0.916454	−0.214532	0.006452	0.041*	
C30	0.9880 (3)	−0.1928 (4)	0.18242 (13)	0.0203 (8)	
C31	1.0439 (4)	−0.3196 (4)	0.17370 (15)	0.0272 (9)	
H31	1.013838	−0.370665	0.146990	0.033*	
C32	1.1392 (4)	−0.3713 (5)	0.20210 (16)	0.0311 (10)	
H32	1.175725	−0.454914	0.194121	0.037*	
C33	1.1835 (4)	−0.3025 (5)	0.24272 (16)	0.0309 (10)	
H33	1.249963	−0.338361	0.262385	0.037*	
C34	1.1293 (4)	−0.1827 (5)	0.25362 (15)	0.0267 (9)	
H34	1.157426	−0.137272	0.281780	0.032*	
C35	1.0322 (3)	−0.1236 (4)	0.22427 (14)	0.0207 (8)	
C36	0.9814 (3)	0.0058 (4)	0.23845 (13)	0.0191 (8)	
C38	0.9638 (5)	0.1729 (5)	0.29017 (16)	0.0433 (13)	
H38A	1.024830	0.244297	0.299462	0.052*	
H38B	0.908083	0.162360	0.315504	0.052*	
C39	0.8918 (4)	0.2105 (4)	0.24440 (13)	0.0230 (8)	
C41	0.9493 (5)	0.3302 (4)	0.22070 (15)	0.0340 (11)	
H41A	0.906794	0.344554	0.189583	0.051*	
H41B	1.036097	0.311041	0.217469	0.051*	
H41C	0.942135	0.412284	0.239752	0.051*	
C42	0.7588 (4)	0.2413 (6)	0.2513 (2)	0.0558 (17)	
H42A	0.719510	0.159755	0.262947	0.084*	
H42B	0.716179	0.269040	0.221383	0.084*	
H42C	0.754580	0.315123	0.274210	0.084*	
N1	1.0186 (3)	0.1251 (3)	0.11733 (12)	0.0216 (7)	
H1	1.065691	0.052590	0.117092	0.026*	
N12	0.8051 (3)	0.2885 (3)	0.09365 (11)	0.0186 (7)	
N15	0.6330 (3)	0.0598 (3)	0.15501 (11)	0.0215 (7)	
H15	0.619157	0.118443	0.177229	0.026*	
N26	0.7256 (3)	−0.0444 (3)	0.06940 (11)	0.0177 (7)	
N29	0.8955 (3)	−0.1416 (3)	0.15281 (11)	0.0190 (7)	
H29	0.891440	−0.201964	0.130248	0.023*	
N40	0.9045 (3)	0.0857 (3)	0.21535 (11)	0.0176 (7)	
O9	0.8521 (3)	0.4775 (3)	0.05403 (12)	0.0371 (8)	
O23	0.5856 (3)	−0.1705 (3)	0.02681 (9)	0.0294 (7)	
O37	1.0238 (3)	0.0466 (3)	0.28185 (10)	0.0272 (7)	
Yb1	0.82829 (2)	0.06815 (2)	0.13487 (2)	0.01617 (6)	

### Tris[2-(4,4-dimethyl-2-oxazolin-2-yl)anilinido]ytterbium(III) (YbH-L133) Atomic displacement parameters (Å^2^)

**Table:**

	*U*^11^	*U*^22^	*U*^33^	*U*^12^	*U*^13^	*U*^23^
C2	0.0199 (19)	0.026 (2)	0.0154 (19)	−0.0011 (16)	0.0074 (15)	−0.0038 (15)
C3	0.019 (2)	0.034 (2)	0.027 (2)	0.0026 (17)	0.0050 (16)	−0.0058 (18)
C4	0.022 (2)	0.038 (3)	0.036 (3)	−0.0077 (19)	0.0092 (19)	−0.013 (2)
C5	0.031 (2)	0.028 (2)	0.046 (3)	−0.0154 (19)	0.019 (2)	−0.012 (2)
C6	0.030 (2)	0.022 (2)	0.034 (2)	−0.0019 (17)	0.0083 (19)	−0.0054 (18)
C7	0.027 (2)	0.0137 (18)	0.020 (2)	−0.0045 (16)	0.0076 (16)	−0.0064 (15)
C8	0.027 (2)	0.024 (2)	0.021 (2)	0.0000 (17)	0.0040 (17)	0.0031 (17)
C10	0.028 (2)	0.036 (3)	0.060 (3)	−0.002 (2)	−0.004 (2)	0.020 (2)
C11	0.021 (2)	0.020 (2)	0.037 (2)	0.0027 (17)	0.0009 (18)	0.0040 (18)
C13	0.027 (2)	0.041 (3)	0.051 (3)	0.010 (2)	−0.007 (2)	−0.019 (2)
C14	0.021 (2)	0.027 (2)	0.031 (2)	0.0041 (17)	−0.0044 (17)	−0.0010 (18)
C16	0.0172 (18)	0.0188 (19)	0.0175 (19)	0.0030 (15)	0.0036 (15)	0.0049 (15)
C17	0.0190 (19)	0.023 (2)	0.023 (2)	0.0040 (16)	0.0076 (16)	0.0048 (16)
C18	0.0184 (19)	0.029 (2)	0.027 (2)	0.0004 (17)	0.0082 (16)	0.0060 (18)
C19	0.019 (2)	0.029 (2)	0.033 (2)	−0.0079 (17)	0.0036 (17)	0.0047 (18)
C20	0.024 (2)	0.026 (2)	0.022 (2)	−0.0082 (17)	0.0027 (16)	−0.0005 (17)
C21	0.0153 (17)	0.0195 (19)	0.0188 (19)	−0.0020 (15)	0.0025 (14)	0.0048 (15)
C22	0.021 (2)	0.022 (2)	0.0148 (18)	−0.0023 (15)	−0.0011 (15)	−0.0010 (15)
C24	0.026 (2)	0.045 (3)	0.020 (2)	−0.011 (2)	0.0099 (17)	−0.0054 (19)
C25	0.0186 (18)	0.030 (2)	0.0156 (18)	−0.0018 (17)	0.0047 (14)	−0.0025 (16)
C27	0.022 (2)	0.032 (2)	0.021 (2)	0.0011 (17)	0.0077 (16)	0.0048 (17)
C28	0.029 (2)	0.026 (2)	0.027 (2)	0.0031 (18)	0.0125 (18)	−0.0013 (18)
C30	0.0196 (19)	0.021 (2)	0.0220 (19)	−0.0042 (16)	0.0103 (15)	0.0046 (16)
C31	0.029 (2)	0.023 (2)	0.032 (2)	0.0011 (17)	0.0144 (18)	0.0046 (18)
C32	0.028 (2)	0.026 (2)	0.042 (3)	0.0071 (18)	0.019 (2)	0.015 (2)
C33	0.020 (2)	0.035 (2)	0.039 (3)	0.0008 (18)	0.0069 (18)	0.021 (2)
C34	0.0170 (19)	0.038 (3)	0.025 (2)	−0.0059 (18)	0.0050 (16)	0.0090 (19)
C35	0.0177 (19)	0.022 (2)	0.024 (2)	−0.0038 (15)	0.0083 (16)	0.0059 (16)
C36	0.0165 (18)	0.028 (2)	0.0130 (18)	−0.0071 (16)	0.0037 (14)	0.0002 (16)
C38	0.082 (4)	0.021 (2)	0.025 (2)	−0.004 (2)	−0.006 (2)	−0.0028 (19)
C39	0.023 (2)	0.026 (2)	0.020 (2)	−0.0036 (17)	0.0037 (16)	−0.0083 (17)
C41	0.054 (3)	0.022 (2)	0.026 (2)	−0.004 (2)	−0.001 (2)	−0.0008 (18)
C42	0.032 (3)	0.072 (4)	0.066 (4)	−0.006 (3)	0.018 (3)	−0.048 (3)
N1	0.0143 (16)	0.0217 (17)	0.0293 (19)	0.0022 (13)	0.0052 (14)	0.0059 (14)
N12	0.0147 (15)	0.0190 (16)	0.0220 (17)	−0.0015 (13)	0.0012 (12)	0.0013 (13)
N15	0.0207 (16)	0.0261 (18)	0.0183 (16)	−0.0050 (14)	0.0057 (13)	−0.0072 (14)
N26	0.0175 (16)	0.0219 (18)	0.0143 (15)	0.0006 (13)	0.0040 (12)	−0.0007 (13)
N29	0.0249 (17)	0.0176 (16)	0.0149 (16)	0.0008 (13)	0.0035 (13)	−0.0014 (13)
N40	0.0203 (16)	0.0175 (16)	0.0154 (15)	−0.0047 (13)	0.0038 (12)	−0.0019 (13)
O9	0.0285 (17)	0.0294 (17)	0.053 (2)	−0.0036 (13)	−0.0018 (15)	0.0204 (15)
O23	0.0247 (15)	0.0445 (19)	0.0197 (15)	−0.0152 (13)	0.0070 (12)	−0.0109 (13)
O37	0.0274 (15)	0.0358 (18)	0.0178 (14)	−0.0039 (13)	−0.0018 (12)	−0.0018 (12)
Yb1	0.01452 (8)	0.01806 (9)	0.01624 (9)	−0.00202 (7)	0.00307 (6)	0.00040 (7)

### Tris[2-(4,4-dimethyl-2-oxazolin-2-yl)anilinido]ytterbium(III) (YbH-L133) Geometric parameters (Å, º)

**Table:**

C2—C3	1.423 (5)	C25—C28	1.513 (6)
C2—C7	1.414 (5)	C25—N26	1.514 (5)
C2—N1	1.356 (5)	C27—H27A	0.9800
C3—H3	0.9500	C27—H27B	0.9800
C3—C4	1.358 (6)	C27—H27C	0.9800
C4—H4	0.9500	C28—H28A	0.9800
C4—C5	1.388 (7)	C28—H28B	0.9800
C5—H5	0.9500	C28—H28C	0.9800
C5—C6	1.372 (6)	C30—C31	1.419 (6)
C6—H6	0.9500	C30—C35	1.427 (6)
C6—C7	1.414 (5)	C30—N29	1.361 (5)
C7—C8	1.445 (6)	C31—H31	0.9500
C8—N12	1.303 (5)	C31—C32	1.366 (6)
C8—O9	1.362 (5)	C32—H32	0.9500
C10—H10A	0.9900	C32—C33	1.396 (7)
C10—H10B	0.9900	C33—H33	0.9500
C10—C11	1.526 (6)	C33—C34	1.365 (6)
C10—O9	1.439 (5)	C34—H34	0.9500
C11—C13	1.519 (6)	C34—C35	1.423 (6)
C11—C14	1.512 (6)	C35—C36	1.458 (6)
C11—N12	1.511 (5)	C36—N40	1.292 (5)
C13—H13A	0.9800	C36—O37	1.350 (4)
C13—H13B	0.9800	C38—H38A	0.9900
C13—H13C	0.9800	C38—H38B	0.9900
C14—H14A	0.9800	C38—C39	1.517 (6)
C14—H14B	0.9800	C38—O37	1.433 (5)
C14—H14C	0.9800	C39—C41	1.520 (6)
C16—C17	1.429 (5)	C39—C42	1.516 (6)
C16—C21	1.432 (5)	C39—N40	1.495 (5)
C16—N15	1.345 (5)	C41—H41A	0.9800
C17—H17	0.9500	C41—H41B	0.9800
C17—C18	1.364 (6)	C41—H41C	0.9800
C18—H18	0.9500	C42—H42A	0.9800
C18—C19	1.393 (6)	C42—H42B	0.9800
C19—H19	0.9500	C42—H42C	0.9800
C19—C20	1.371 (5)	N1—H1	0.8800
C20—H20	0.9500	N1—Yb1	2.252 (3)
C20—C21	1.406 (5)	N12—Yb1	2.468 (3)
C21—C22	1.455 (5)	N15—H15	0.8800
C22—N26	1.289 (5)	N15—Yb1	2.260 (3)
C22—O23	1.366 (4)	N26—Yb1	2.376 (3)
C24—H24A	0.9900	N29—H29	0.8749
C24—H24B	0.9900	N29—Yb1	2.234 (3)
C24—C25	1.532 (5)	N40—Yb1	2.390 (3)
C24—O23	1.445 (5)	Yb1—H29	2.7484
C25—C27	1.515 (5)		
			
C7—C2—C3	116.4 (4)	H28B—C28—H28C	109.5
N1—C2—C3	121.8 (4)	C31—C30—C35	116.4 (4)
N1—C2—C7	121.8 (3)	N29—C30—C31	121.6 (4)
C2—C3—H3	118.6	N29—C30—C35	122.0 (4)
C4—C3—C2	122.9 (4)	C30—C31—H31	118.6
C4—C3—H3	118.6	C32—C31—C30	122.8 (4)
C3—C4—H4	119.9	C32—C31—H31	118.6
C3—C4—C5	120.3 (4)	C31—C32—H32	119.6
C5—C4—H4	119.9	C31—C32—C33	120.7 (4)
C4—C5—H5	120.4	C33—C32—H32	119.6
C6—C5—C4	119.2 (4)	C32—C33—H33	120.7
C6—C5—H5	120.4	C34—C33—C32	118.7 (4)
C5—C6—H6	119.2	C34—C33—H33	120.7
C5—C6—C7	121.7 (4)	C33—C34—H34	118.8
C7—C6—H6	119.2	C33—C34—C35	122.3 (4)
C2—C7—C8	122.4 (3)	C35—C34—H34	118.8
C6—C7—C2	119.4 (4)	C30—C35—C36	122.6 (4)
C6—C7—C8	118.2 (4)	C34—C35—C30	119.1 (4)
N12—C8—C7	130.5 (4)	C34—C35—C36	118.4 (4)
N12—C8—O9	115.7 (4)	N40—C36—C35	129.4 (4)
O9—C8—C7	113.7 (3)	N40—C36—O37	116.7 (4)
H10A—C10—H10B	109.0	O37—C36—C35	113.9 (3)
C11—C10—H10A	111.0	H38A—C38—H38B	108.7
C11—C10—H10B	111.0	C39—C38—H38A	110.5
O9—C10—H10A	111.0	C39—C38—H38B	110.5
O9—C10—H10B	111.0	O37—C38—H38A	110.5
O9—C10—C11	103.9 (4)	O37—C38—H38B	110.5
C13—C11—C10	111.4 (4)	O37—C38—C39	106.3 (3)
C14—C11—C10	110.1 (4)	C38—C39—C41	111.7 (4)
C14—C11—C13	111.7 (4)	C42—C39—C38	111.8 (4)
N12—C11—C10	101.8 (3)	C42—C39—C41	110.0 (4)
N12—C11—C13	108.3 (3)	N40—C39—C38	102.4 (3)
N12—C11—C14	113.2 (3)	N40—C39—C41	109.0 (3)
C11—C13—H13A	109.5	N40—C39—C42	111.8 (3)
C11—C13—H13B	109.5	C39—C41—H41A	109.5
C11—C13—H13C	109.5	C39—C41—H41B	109.5
H13A—C13—H13B	109.5	C39—C41—H41C	109.5
H13A—C13—H13C	109.5	H41A—C41—H41B	109.5
H13B—C13—H13C	109.5	H41A—C41—H41C	109.5
C11—C14—H14A	109.5	H41B—C41—H41C	109.5
C11—C14—H14B	109.5	C39—C42—H42A	109.5
C11—C14—H14C	109.5	C39—C42—H42B	109.5
H14A—C14—H14B	109.5	C39—C42—H42C	109.5
H14A—C14—H14C	109.5	H42A—C42—H42B	109.5
H14B—C14—H14C	109.5	H42A—C42—H42C	109.5
C17—C16—C21	115.9 (3)	H42B—C42—H42C	109.5
N15—C16—C17	120.4 (4)	C2—N1—H1	110.7
N15—C16—C21	123.7 (3)	C2—N1—Yb1	138.6 (3)
C16—C17—H17	118.7	Yb1—N1—H1	110.7
C18—C17—C16	122.6 (4)	C8—N12—C11	106.4 (3)
C18—C17—H17	118.7	C8—N12—Yb1	127.8 (3)
C17—C18—H18	119.5	C11—N12—Yb1	125.7 (2)
C17—C18—C19	121.0 (4)	C16—N15—H15	112.5
C19—C18—H18	119.5	C16—N15—Yb1	134.9 (3)
C18—C19—H19	120.8	Yb1—N15—H15	112.5
C20—C19—C18	118.4 (4)	C22—N26—C25	107.9 (3)
C20—C19—H19	120.8	C22—N26—Yb1	127.4 (3)
C19—C20—H20	118.7	C25—N26—Yb1	124.2 (2)
C19—C20—C21	122.7 (4)	C30—N29—H29	101.5
C21—C20—H20	118.7	C30—N29—Yb1	134.3 (3)
C16—C21—C22	122.2 (3)	Yb1—N29—H29	117.4
C20—C21—C16	119.3 (3)	C36—N40—C39	107.5 (3)
C20—C21—C22	118.3 (3)	C36—N40—Yb1	127.6 (3)
N26—C22—C21	130.6 (3)	C39—N40—Yb1	123.6 (2)
N26—C22—O23	115.8 (3)	C8—O9—C10	106.4 (3)
O23—C22—C21	113.6 (3)	C22—O23—C24	106.5 (3)
H24A—C24—H24B	108.9	C36—O37—C38	106.4 (3)
C25—C24—H24A	110.8	N1—Yb1—N12	74.72 (11)
C25—C24—H24B	110.8	N1—Yb1—N15	167.59 (12)
O23—C24—H24A	110.8	N1—Yb1—N26	109.02 (11)
O23—C24—H24B	110.8	N1—Yb1—H29	89.3
O23—C24—C25	104.8 (3)	N1—Yb1—N40	86.61 (11)
C27—C25—C24	111.8 (3)	N12—Yb1—H29	147.2
C28—C25—C24	111.6 (4)	N15—Yb1—N12	95.23 (11)
C28—C25—C27	111.0 (3)	N15—Yb1—N26	77.79 (11)
C28—C25—N26	109.6 (3)	N15—Yb1—H29	103.0
N26—C25—C24	101.6 (3)	N15—Yb1—N40	91.10 (11)
N26—C25—C27	110.9 (3)	N26—Yb1—N12	90.47 (10)
C25—C27—H27A	109.5	N26—Yb1—H29	67.5
C25—C27—H27B	109.5	N26—Yb1—N40	153.87 (10)
C25—C27—H27C	109.5	N29—Yb1—N1	89.28 (12)
H27A—C27—H27B	109.5	N29—Yb1—N12	159.71 (11)
H27A—C27—H27C	109.5	N29—Yb1—N15	102.04 (12)
H27B—C27—H27C	109.5	N29—Yb1—N26	82.94 (11)
C25—C28—H28A	109.5	N29—Yb1—H29	16.4
C25—C28—H28B	109.5	N29—Yb1—N40	76.28 (11)
C25—C28—H28C	109.5	N40—Yb1—N12	114.28 (10)
H28A—C28—H28B	109.5	N40—Yb1—H29	92.7
H28A—C28—H28C	109.5		
			
C2—C3—C4—C5	−2.3 (7)	C30—C35—C36—O37	171.8 (3)
C2—C7—C8—N12	8.6 (7)	C31—C30—C35—C34	1.3 (5)
C2—C7—C8—O9	−174.9 (4)	C31—C30—C35—C36	−178.4 (3)
C3—C2—C7—C6	4.7 (5)	C31—C30—N29—Yb1	−152.8 (3)
C3—C2—C7—C8	−172.0 (4)	C31—C32—C33—C34	0.3 (6)
C3—C2—N1—Yb1	152.0 (3)	C32—C33—C34—C35	−2.1 (6)
C3—C4—C5—C6	2.7 (7)	C33—C34—C35—C30	1.2 (6)
C4—C5—C6—C7	0.7 (7)	C33—C34—C35—C36	−179.0 (4)
C5—C6—C7—C2	−4.5 (6)	C34—C35—C36—N40	171.0 (4)
C5—C6—C7—C8	172.4 (4)	C34—C35—C36—O37	−7.9 (5)
C6—C7—C8—N12	−168.2 (4)	C35—C30—C31—C32	−3.2 (6)
C6—C7—C8—O9	8.3 (5)	C35—C30—N29—Yb1	28.5 (5)
C7—C2—C3—C4	−1.5 (6)	C35—C36—N40—C39	−175.0 (4)
C7—C2—N1—Yb1	−27.8 (6)	C35—C36—N40—Yb1	−7.7 (6)
C7—C8—N12—C11	171.3 (4)	C35—C36—O37—C38	−179.2 (4)
C7—C8—N12—Yb1	−7.9 (6)	C38—C39—N40—C36	−7.3 (4)
C7—C8—O9—C10	172.1 (4)	C38—C39—N40—Yb1	−175.2 (3)
C10—C11—N12—C8	17.7 (4)	C39—C38—O37—C36	−6.4 (5)
C10—C11—N12—Yb1	−163.1 (3)	C41—C39—N40—C36	111.1 (4)
C11—C10—O9—C8	21.3 (5)	C41—C39—N40—Yb1	−56.9 (4)
C13—C11—N12—C8	−99.8 (4)	C42—C39—N40—C36	−127.1 (4)
C13—C11—N12—Yb1	79.4 (4)	C42—C39—N40—Yb1	64.9 (4)
C14—C11—N12—C8	135.8 (4)	N1—C2—C3—C4	178.7 (4)
C14—C11—N12—Yb1	−45.0 (5)	N1—C2—C7—C6	−175.5 (4)
C16—C17—C18—C19	−1.1 (6)	N1—C2—C7—C8	7.8 (6)
C16—C21—C22—N26	8.4 (6)	N12—C8—O9—C10	−10.8 (5)
C16—C21—C22—O23	−171.1 (3)	N15—C16—C17—C18	−177.3 (4)
C17—C16—C21—C20	−2.6 (5)	N15—C16—C21—C20	177.2 (4)
C17—C16—C21—C22	173.4 (3)	N15—C16—C21—C22	−6.8 (6)
C17—C16—N15—Yb1	168.1 (3)	N26—C22—O23—C24	−11.2 (5)
C17—C18—C19—C20	−0.4 (6)	N29—C30—C31—C32	178.0 (4)
C18—C19—C20—C21	0.3 (6)	N29—C30—C35—C34	−179.9 (3)
C19—C20—C21—C16	1.3 (6)	N29—C30—C35—C36	0.4 (6)
C19—C20—C21—C22	−174.8 (4)	N40—C36—O37—C38	1.8 (5)
C20—C21—C22—N26	−175.7 (4)	O9—C8—N12—C11	−5.1 (5)
C20—C21—C22—O23	4.9 (5)	O9—C8—N12—Yb1	175.7 (3)
C21—C16—C17—C18	2.6 (6)	O9—C10—C11—C13	91.9 (4)
C21—C16—N15—Yb1	−11.7 (6)	O9—C10—C11—C14	−143.6 (4)
C21—C22—N26—C25	179.8 (4)	O9—C10—C11—N12	−23.3 (5)
C21—C22—N26—Yb1	7.3 (6)	O23—C22—N26—C25	−0.8 (5)
C21—C22—O23—C24	168.3 (3)	O23—C22—N26—Yb1	−173.3 (2)
C24—C25—N26—C22	11.5 (4)	O23—C24—C25—C27	−135.6 (4)
C24—C25—N26—Yb1	−175.7 (3)	O23—C24—C25—C28	99.4 (4)
C25—C24—O23—C22	17.6 (4)	O23—C24—C25—N26	−17.3 (4)
C27—C25—N26—C22	130.5 (3)	O37—C36—N40—C39	3.8 (4)
C27—C25—N26—Yb1	−56.8 (4)	O37—C36—N40—Yb1	171.2 (2)
C28—C25—N26—C22	−106.7 (4)	O37—C38—C39—C41	−108.3 (4)
C28—C25—N26—Yb1	66.1 (4)	O37—C38—C39—C42	128.0 (4)
C30—C31—C32—C33	2.5 (6)	O37—C38—C39—N40	8.2 (5)
C30—C35—C36—N40	−9.3 (6)		
